# Risk factors associated with suicidal ideation among cancer patients: a systematic review and meta-analysis

**DOI:** 10.3389/fpsyg.2023.1287290

**Published:** 2024-01-08

**Authors:** Jie Chen, Zhiheng Ping, Deying Hu, Jiaqing Wang, Yilan Liu

**Affiliations:** ^1^Department of Nursing, Union Hospital, Tongji Medical College, Huazhong University of Science and Technology, Wuhan, China; ^2^School of Nursing, Tongji Medical College, Huazhong University of Science and Technology, Wuhan, China; ^3^Cancer Center, Union Hospital, Tongji Medical College, Huazhong University of Science and Technology, Wuhan, China

**Keywords:** suicidal ideation, risk factors, cancer patients, systematic review, meta-analysis

## Abstract

**Objective:**

The objective of this review was to provide a comprehensive summary and analysis of the risk factors associated with suicidal ideation among cancer patients.

**Methods:**

This review adhered to the PICO/S framework and guidelines outlined in the Preferred Reporting Items for Systematic Review and Meta-Analysis (PRISMA) framework (PROSPERO CRD42023433639). We searched Web of Science, PubMed, Embase, Scopus, PsycINFO, and Cochrane Library from the establishment date of the databases until June 9, 2023 for observational studies that reveal risk factors associated with suicidal ideation among cancer patients. Software Review Manager 5 (vision 5.4) was used for Meta-analyses.

**Results:**

4,921 studies were obtained through the search of the databases, 40 of which were eligible. Meta-analysis revealed that suicidal ideation in cancer patients was significantly associated with marital status, living alone, post-traumatic stress disorder (PTSD), panic disorder, education, psychiatric illness history, social functioning, childhood adversity experience, financial problems, pain, depression, demoralization, vomiting, residence and anxiety.

**Conclusion:**

Being unmarried, living alone, less educated, living in rural, financial problems, pain, vomiting, PTSD, psychiatric illness history, lower social functioning, childhood adversity experience, anxiety, depression, demoralization, panic disorder were risk factors for suicidal ideation among cancer patients. This review provided evidence-based information for identifying and reducing the risk of suicide in cancer survivors.

**Systematic review registration**: https://www.crd.york.ac.uk/PROSPERO/, CRD42023433639.

## 1 Introduction

Suicide is a critical public health concern with significant global implications, leading to a substantial increase in mortality rates around the world. The World Health Organization (WHO) estimates that approximately 703,000 individuals die by suicide each year on a global scale (World Health Organization, 2021). Furthermore, experiences related to suicide, such as suicidal ideation, suicide attempts, suicide planning, and impulsive thoughts of suicide, can have a profound and detrimental psychological effect on both the individual and those in close proximity to them (Levi-Belz et al., 2019). According to the World Health Organization’s (WHO) 2019 estimates, cancer is identified as the primary or secondary cause of mortality before the age of 70 in 112 out of 183 countries, while ranking as the third or fourth leading cause in a further 23 countries (Sung et al., 2021). With the progress of medical science and the evolution of treatment approaches, an increasing number of cancer patients succumb to non-cancer-related causes (Yang et al., 2021). They endure significant physical and psychological stress. The rapid and devastating nature of suicide has made it a prominent contributor to mortality in cancer patients. The emotional upheaval triggered by a cancer diagnosis, the adverse effects of treatment, societal stigmatization leading to social isolation, and the financial burden of exorbitant medical expenses all contribute to an increased risk of suicidal tendencies among individuals grappling with cancer (Du et al., 2020). In daily life, patients are more prone to experiencing negative emotions due to their heightened attention toward these adverse changes, thereby amplifying the psychological impact on patients and intensifying social demands (Fingeret et al., 2010). For instance, body image alterations in cancer patients, primarily resulting from surgical procedures and long-term chemotherapy treatments, may engender feelings of shame regarding their physical appearance and difficulties in social interactions with friends and partners, ultimately contributing to the emergence of suicidal ideation (Covrig et al., 2021).

The prevalence of suicidal ideation in this particular population varies widely, ranging from 0.7 to 46.3% (Kolva et al., 2020). A recent meta-analysis indicated that the overall prevalence of suicidal ideation among cancer patients in mainland China was 24.95% (Ding et al., 2023). Furthermore, certain subsets of cancer patients experienced even higher rates, with the incidence of suicidal ideation reaching as high as 34.7% (Choi et al., 2014). Suicidal ideation encompasses a broad spectrum of thoughts, wishes, and concerns related to death and suicide. Suicidal ideation is considered to be one of the most important warning signs of suicidal behavior, and early detection and appropriate management of suicidal ideation is very important. According to the cumulative evidence, several studies have reported a range of risk factors for suicidal ideation in cancer patients. For example, general demographic risk factors include age (Akechi et al., 2000; Walker et al., 2008; Ernst et al., 2022), gender (Hagezom et al., 2021; Ma et al., 2022; Tang et al., 2022), religion (Spencer et al., 2012; Trevino et al., 2014b), education level (Zhou et al., 2020; Tang et al., 2022) and so on. The disease-related factors included pain (Akechi et al., 2002; Walker et al., 2008; Tang et al., 2016), cancer stage (Hagezom et al., 2021; Yu et al., 2023) and time since diagnosis (Zhou et al., 2020; Yu et al., 2023). Psychological factors include depression (Chang et al., 2019; Akechi et al., 2020; Xu et al., 2020), anxiety (Madeira et al., 2011; Hagezom et al., 2021) and hopelessness (Costantini et al., 2014; Zhang et al., 2017). Social factors encompass social support (Tang et al., 2016), social functioning (Jung and Yun, 2022) and so on. Based on previous research into risk factors for suicidal ideation, researchers have proposed various psychological interventions, including mindfulness meditation (Boyle et al., 2017) and music intervention (Trigueros-Murillo et al., 2023). However, the impact of some risk factors on suicidal ideation has been reported differently in studies making it challenging to further investigate interventions for cancer patients experiencing such thoughts. For instance, there are conflicting findings regarding the likelihood of suicidal ideation between female and male cancer patients. Some studies indicate a higher prevalence of suicidal ideation among female cancer patients, while others report the opposite trend (Madeira et al., 2011; Katayama et al., 2023). Furthermore, variations in findings exist across different countries or regions. In Italy, certain scholars have found no significant correlation between chemotherapy and suicidal ideation in cancer patients (Costantini et al., 2014). Conversely, studies conducted in China have indicated a substantial likelihood of chemotherapy being a risk factor for suicidal ideation among cancer patients (Tang et al., 2016). However, there is a lack of comprehensive meta-analyses or systematic reviews that have synthesized these factors. Currently, several studies are being conducted to investigate the risk factors associated with suicidal ideation in cancer patients. Nevertheless, these studies have reported inconsistent findings, and a comprehensive analysis of the impact of specific risk factors on suicidal ideation is still lacking. Hence, the purpose of this systematic review and meta-analysis is to concisely summarize the risk factors that contribute to suicidal ideation among cancer patients. Additionally, it aims to analyze the extent of the impact of these risk factors and offer evidence-based insights to identify suicide indicators early and develop mitigation strategies specifically for cancer survivors.

## 2 Methods

We adhered to the guidelines of the Preferred Reporting Items for Systematic Reviews and Meta-Analyses (PRISMA) to carry out our systematic review and meta-analysis. The review protocol has been registered in PROSPERO (registration number CRD42023433639).

We adhered to the PICO/S framework, as follows:

Population: cancer patients.

Exposure: any potential risk factor which may have an impact on suicidal ideation of cancer patients.

Comparison: cancer patients without suicidal ideation.

Outcome: suicidal ideation.

Study type: cross-sectional, case–control or cohort study.

### 2.1 Search strategy

Two researchers (JC and ZH) independently conducted comprehensive literature searches in major electronic databases, including Web of Science, PubMed, Embase, Scopus, PsycINFO, and the Cochrane Library. The searches were conducted from the inception of the databases until June 9, 2023, in order to identify observational studies that investigate risk factors associated with suicidal ideation among cancer patients. The search strategy employed a combination of subject headings and keywords. The search terms included: (“Neoplasm” [MeSH Terms] OR “cancer” OR “tumor”) AND (“suicidal ideation”) AND (“risk factors” OR “associated factors” OR “related factors” OR “influenc* factors” OR “risk*” OR “factors”). The reference lists of previously relevant publications were searched to identify relevant studies and guarantee the comprehensiveness and accuracy of the retrieval strategy utilized in this study.

### 2.2 Inclusion and exclusion criteria

The inclusion criteria included: (1) population (P): cancer patients; (2) exposure factors (E): any potential risk factor which may have an impact on suicidal ideation of cancer patients; (3) outcome (O): suicidal ideation, which is necessary to adhere to internationally recognized suicidal ideation assessment scales; (4) study type: observational studies including case–control, cohort study, or cross-sectional design; (5) English literature that has been published online.

The exclusion criteria included: (1) books, case reports, editorials, comments, conference papers, any type of review, or letters; (2) the study participants are underage (<18 years old); (3) duplicate literature or data; (4) studies not providing full text or specific data or only providing the statistical conclusion.

### 2.3 Data extraction

The study selection process and extracted data are as follows: (1) EndNoteX9 software was used to manage exported search results and remove duplicates. (2) Two authors (JC and ZH) independently screened the titles and abstracts and evaluated the applicability of the criteria. At this stage, only studies that met the minimum quality criteria were selected. (3) They carefully reviewed the chosen studies in their entirety to assess their compatibility with the predefined eligibility criteria. (4) Both reviewers examined 10% of the studies included by the other reviewer to determine eligibility. The task of resolving disagreements was assigned to the third author, DY. (5) Two researchers (JC and ZH) extracted data from eligible studies and recorded it in a spreadsheet. The data included the first author’s name, country of the study population, year of publication, age, sample size, type of cancer, prevalence of suicidal ideation and study design. A quality assessment was also be carried out at this stage.

### 2.4 Quality assessment

The quality of the included studies was independently assessed by two researchers (JC and ZH), and any disagreements were resolved through discussion with an additional reviewer (DY). To evaluate the included case–control and cohort studies, we utilized the Newcastle-Ottawa scale (Stang, 2010). The scale comprises 8 items, with scores ranging from 0 to 3 denoting low quality, 4 to 6 indicating moderate quality, and 7 to 9 representing high quality. We evaluated the included cross-sectional studies using the checklist provided by the U.S. Agency for Healthcare Research and Quality (AHRQ) (Schünemann et al., 2008). The checklist is comprised of 11 items, each with answer options of “yes,” “no,” or “unclear.” A response of “yes” is assigned 1 point, while “unclear” or “no” receive 0 points. A total score falling within the range of 8–11 indicates high quality, 4–7 suggests moderate quality, and 0–3 reflects low quality.

### 2.5 Statistical analysis

Data analysis was conducted using Software Review Manager 5 (version 5.4). The heterogeneity test between studies was performed based on *I*^2^ and *p*-values. If the results showed *p* ≥ 0.05 and/or *I*^2^ ≤ 50%, it indicated homogeneity among the studies and a fixed-effect model was utilized. Conversely, if heterogeneity was observed, a random-effects model was employed (Higgins et al., 2003). To assess the stability of the results, sensitivity analysis was conducted by excluding each study individually. The presence of publication bias was analyzed using the funnel plot method. The effect of count data was quantified using the odds ratio (OR) and the corresponding 95% confidence interval (CI). And the effect of continuous data was represented by the standardized mean difference (MD) along with its 95% confidence interval (CI). A significance level of *p* < 0.05 (two-tailed) was deemed statistically significant. Risk factors with frequencies less than 2 or variables with inconsistent classifications or different data types were not included in the meta-analysis. For some factors, if the number of studies was only 2 and there was heterogeneity, meta-analysis would not be performed.

## 3 Results

### 3.1 Study search results

**Figure 1:**
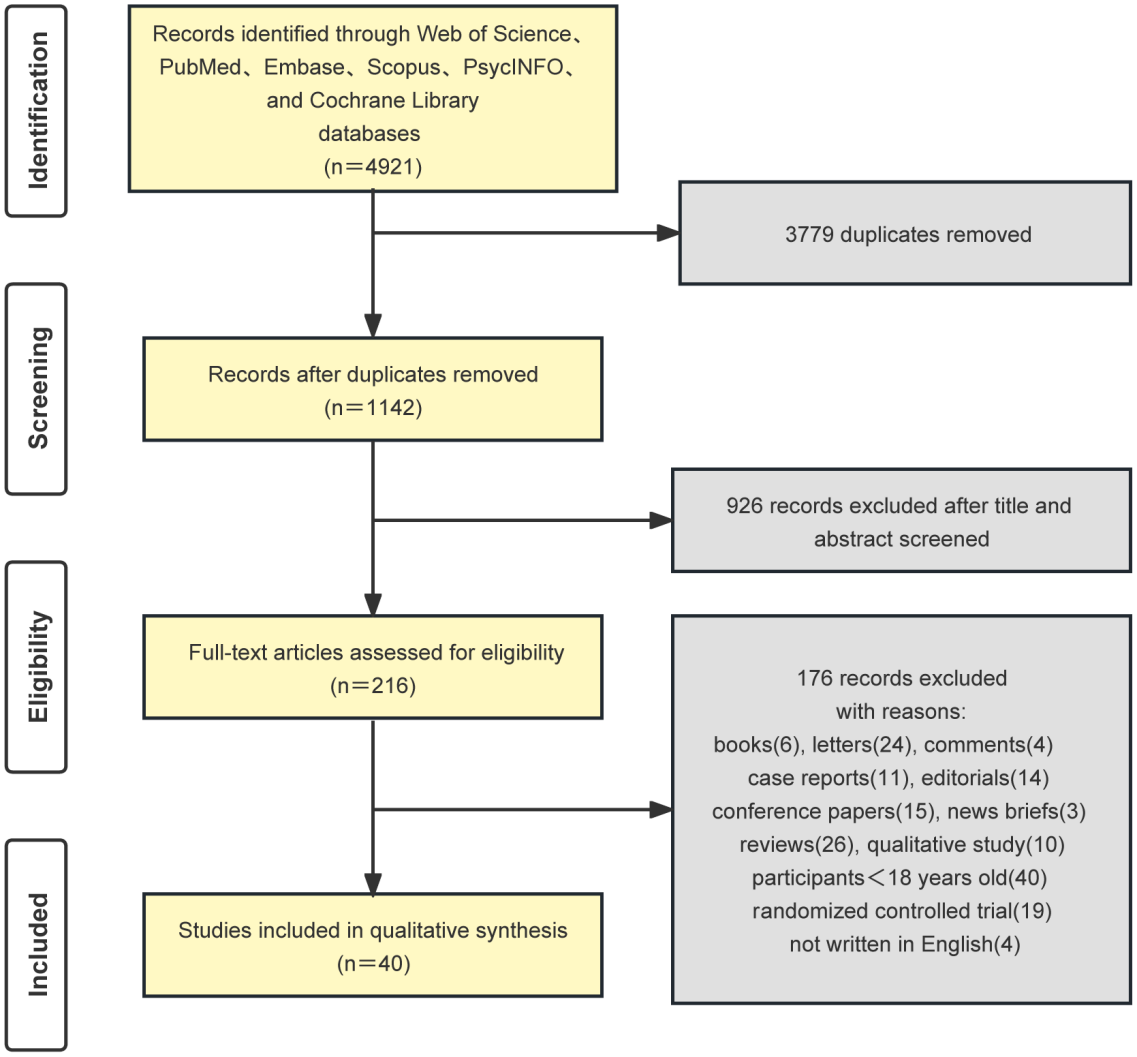
The flowchart of the study selection process.

According to the searching strategy, a total of 4,921 studies were retrieved from the database initially. Following the exclusion of duplicate articles, 1,142 records were screened out. By reading the abstract and title, 926 studies that were obviously inconsistent with the theme were excluded, and 216 were included in the preliminary screening. Following the application of the inclusion and exclusion criteria to the full text, a total of 40 studies that met the conditions were included. Figure 1 displays the flowchart of the study selection process.

### 3.2 Characteristics of the studies included

The selected studies were published from 2000 to 2023. The study included participants from thirteen countries, namely China, the United States, Japan, South Korea, the United Kingdom, Italy, Germany, Portugal, Lithuania, Ethiopia, Canada, Australia, and Spain, with sample sizes ranging from 60 to 382,266. The prevalence of suicidal ideation among cancer patients ranged from 0.27 to 53.3%. Out of the 40 included studies, the majority (30) were cross-sectional in design. 9 were cohort studies and 1 was case–control study. The 30 cross-sectional studies were assessed for quality, with scores ranging from 4 to 10 and an average score of 6.70, indicating a moderate quality level (6.70 out of 11). The 9 cohort studies scored from 5 to 8, with an average score of 5.89, and were of moderate quality. The score of the case–control study was 7, which indicated high quality. The basic characteristics and quality evaluation of the studies were demonstrated in Table 1.

**Table 1:** The basic characteristics and quality evaluation of the studies included.

Study ID	Study (Author, Year)	Country	Study Design	Cancer type	Sample Size (female, %)	Age, Mean ± (SD) or median (interquartile range [IQR])	Prevalence of SI (%)	AHRQ	NOS
1	Xu et al. (2020)	China	Cross-Sectional	Mixed	544 (51.8)	59.9 (NA)	26.3	9	
2	Lee et al. (2022)	South Korea	Cross-sectional	Urologic cancer	60 (11.67)	NA (NA)	NA	5	
3	Walker et al. (2008)	United Kingdom	Cross-sectional	Mixed	2,924 (64)	60.3 (NA)	7.8	8	
4	Spencer et al. (2012)	America	Cross-sectional	Mixed	700 (48.6)	59.1 (13.1)	8.9	10	
5	Madeira et al. (2011)	Portugal	Cross-sectional	Mixed	130 (73.1)	NA	35	4	
6	Lee et al. (2014)	South Korea	Case–control	Mixed	2,472 (47.1)	NA	NA		7
7	Tang et al. (2016)	China	Cross-sectional	Gynecological cancer	579 (100)	18-78y	18.1	5	
8	Kazlauskiene et al. (2022)	Lithuania	Prospective cohort	Breast cancer	Prior to surgery: 421 (100), 1 year after surgery: 188 (100)	NA (NA)	Prior to surgery:4.3, 1 year after surgery: 12.8		5
9	Akechi et al. (2020)	Japan	Cross-sectional	Multiple myeloma	79 (about 50)	66.0 (11.0)	12.6	6	
10	Chang et al. (2019)	China, Taiwan	Retrospective cohort	Head and neck cancer	286 (3.8)	no SI: 57.3 (12.0), SI: 54.1 (10.0)	9.4		5
11	Hagezom et al. (2021)	Ethiopia	Cross-sectional	Mixed	410 (51)	46.7 (12.9)	28.5	5	
12	Akechi et al. (2010)	Japan	Retrospective cohort	Mixed	728 (54.8)	58.0 (12.0)	40		5
13	Jung and Yun (2022)	South Korea	Cross-sectional	Mixed	612 (58.5)	55.7 (NA)	8.1	7	
14	Stanbouly et al. (2023)	America	Retrospective cohort	Head and neck cancer	29,231 (29)	NA	0.35		6
15	Hyer et al. (2021)	America	Retrospective cohort	Mixed	211,092 (50.6)	74.0 (NA)	0.27		6
16	Trevino et al. (2014b)	America	Cross-sectional	Mixed	603 (51.2)	59.4 (13.2)	26.2	9	
17	Trevino et al. (2014a)	America	Cross-sectional	Mixed	93 (68.8)	33.3 (5.5)	22.6	8	
18	Akechi et al. (2002)	Japan	Prospective cohort	Lung cancer	89 (28)	61.0 (9.0)	15		7
19	Henry et al. (2018)	Canada	Prospective cohort	Head and neck cancer	223 (30.9)	62.9 (11.7)	15.7		6
20	Costantini et al. (2014)	Italy	Cross-sectional	Mixed	136 (83.1)	51.0 (12.4)	18	9	
21	Xu et al. (2019)	China	Cross-sectional	Mixed	303 (47.5)	NA	14.5	9	
22	Luo et al. (2022)	China	Cross-sectional	Mixed	820 (40.6)	57.0 (NA)	26	5	
23	Ernst et al. (2020)	Germany	Cross-sectional	Mixed	916 (44.2)	34.6 (5.5)	8	6	
24	Zhong et al. (2017)	China	Cross-sectional	Mixed	517 (50.7)	59.7 (11.7)	15.3	8	
25	Johnson et al. (2020)	America	Cross-sectional	Mixed	175 (22.3)	61.8 (10.2)	25.1	6	
26	Ma et al. (2022)	China	Cross-sectional	Mixed	5,670 (47.5)	50.5 (13.2)	13.3	6	
27	Zhang et al. (2017)	China	Cross-sectional	Stomach cancer	151 (20.5)	no SI: 55.2 (12.4), SI: 54.9 (11.4)	17.2	6	
28	Tang et al. (2022)	China	Cross-sectional	Mixed	2,930 (41.3)	56.9 (NA)	13.1	7	
29	Akechi et al. (2000)	Japan	Cross-sectional	Mixed	114 (50)	58.0 (11.0)	53.3	5	
30	Zhang et al. (2020)	China	Cross-sectional	Mixed	603 (90)	47.7 (11.5)	15.1	6	
31	Pranckeviciene et al. (2016)	Austria	Prospective cohort	Brain tumors	211 (70)	55.9 (15.4)	6		5
32	Sauer et al. (2022)	Germany	Cross-sectional	Mixed	4,372 (57.7)	Women: 55.6 (13.1), man: 60.2 (12.5)	14.3	6	
33	Recklitis et al. (2014)	America	Cross-sectional	Prostate cancer	693 (0)	67.1 (1.7)	12.4	7	
34	Walker et al. (2022)	United Kingdom	Cross-sectional	Mixed	2,217 (80)	60.0 (NA)	9	5	
35	Akechi et al. (2004)	Japan	Prospective cohort	Mixed	140 (34)	61.0 (10.0)	8.6		8
36	Diaz-Frutos et al. (2016)	Spanish	Cross-sectional	Mixed	202 (56.9)	NA	25.2	8	
37	Choi et al. (2014)	South Korea	Cross-sectional	Stomach cancer	378 (27)	no SI: 54.8 (10.6), SI: 53.0 (10.2)	34.7	7	
38	Zhou et al. (2020)	China	Cross-sectional	Mixed	357 (50.7)	NA	15.4	6	
39	Yu et al. (2023)	China	Cross-sectional	Lung cancer	366 (29.8)	NA (NA)	22.7	7	
40	Katayama et al. (2023)	America	Cross-sectional	Stomach, liver, pancreas, and colorectal cancers	382,266 (47.6)	Median age: 72y (IQR: 65-80y)	0.4	5	

AHRQ, Agency for Healthcare Research and Quality; NOS, Newcastle-Ottawa Scale; NA, not available; SI, suicidal ideation.

### 3.3 Description of the potential risk factors

Table 2 presents the identification of 122 potential risk factors associated with suicidal ideation in cancer patients. The most common risk factors included depression, anxiety, pain, age, marital status and so on. Risk factors with frequencies less than 2 were not included in the meta-analysis. Factors such as self-efficacy, time since cancer diagnosis, social support, income, alcohol consumption, existential well-being, self-rated economic status, physical function, tumor site, age (count data), fatigue, body image, global health status and number of cancer treatment regimens were not included in the meta-analysis due to inconsistent classifications or different data types. For some factors, if the number of studies was only 2 and there was heterogeneity, meta-analysis would not be performed.

**Table 2:** Potential risk factors associated with suicidal ideation in cancer patients.

Risk factors/Study ID	1	2	3	4	5	6	7	8	9	10	11	12	13	14	15	16	17	18	19	20	21	22	23	24	25	26	27	28	29	30	31	32	33	34	35	36	37	38	39	40
Age			√											√												√			√	√		√		√	√	√				√
Anxiety					√						√			√	√						√	√		√		√				√	√	√	√			√	√			
Appetite loss																												√									√			
Avoidant personality																																				√				
Adjustment disorder					√																																			
Alcohol consumption														√									√																	
Acceptance-resignation							√																																	
Affordability of medical cost																																						√		
Avoidance coping: self-blame																			√																					
Avoidance coping: substance use																			√																					
A relative or friend who suicide																								√																
Body Image																																				√	√			
Burden on others																																			√					
Bipolar/schizophrenia															√																		√			√				
Borderline personality																																				√				
Behavioral disengagement																√																								
Constipation																																					√			
Cancer stage							√				√								√			√																√	√	√
Coping strain																											√													
Confrontation																																						√		
Comorbidity																																					√			
Chemotherapy						√	√													√																				√
Cognitive function																																				√				
CNS tumor diagnosis																							√																	
Confidence in treatment																																						√		
Childhood adversity experience																			√											√										
Diarrhea																																					√			
Dyspnea																																					√			
Dysphoria										√																														
Dysthymia					√																																			
Dry mouth																																					√			
Drowsiness																												√												
Depression	√	√			√		√		√	√				√	√					√	√	√		√	√	√			√	√			√			√		√		
Disease status			√																																					
Demoralization																					√	√																		
Daily exercise time																														√										
Distress thermometer										√																														
Dependent personality																																				√				
Difficulty in remembering																												√												
Education																												√										√		
Eating restriction																																					√			
Emotional function																																				√				
Employment status	√							√																		√							√			√				
Existential well-being																			√																		√			
Fatigue																																			√		√			
Fear of recurrence																														√										
Financial problems																						√															√			
Family adaptability and cohesion																																						√		
Gender					√						√															√		√											√	√
Global health status																																				√	√			
Hostility																										√														
Hair loss																																					√			
Headaches																							√																	
Hopelessness					√														√	√							√									√				
Health insurance														√																										
Hospitalized times																																							√	
Income						√																				√											√			
International Personality Disorders																																				√				
Loneliness																							√																	
Living alone																			√			√																		
Life worthwhile													√																											
Living with loved ones													√																											
Lifetime suicidal ideation																			√																					
Metastasis	√											√				√								√		√		√	√											
Marital status	√		√						√													√	√			√				√										√
Mental disorder history																										√														√
Mental health treatment																																	√							
Mental component score																																	√							
Mental healthcare providers																																								√
Nausea																												√												
Neuroticism																			√																					
Nicotine dependence														√																										
Negative religious coping																√																								
Number of physical symptoms																√	√																					√	√	
Number of cancer treatment																																						√		√
Pain			√		√		√							√				√						√				√		√	√		√		√					
Palliative care																																				√				
Panic disorder				√	√																																			
Physical function																																	√			√				
Pain management																√																								
Parkinson’s disease																√																								
Performance status												√				√	√							√					√						√					
Psychological strain																											√													
Physical comorbidity																														√										
Past suicide attempts																							√		√															
Primary care providers							√																																	
Previous cancer history										√																														
Psychiatric illness history					√											√	√	√	√					√							√				√					
Psychomotor retardation										√																														
Post-traumatic stress disorder				√												√	√								√															
Race																√																								√
Religion				√												√						√		√															√	
Residence								√														√																√		
Recurrence						√						√																	√											
Role function																																				√				
Radiotherapy																														√										√
Role limitations																															√									
Stigma											√																													
Sadness																												√												
Smoking						√																						√									√		√	
Site of HNC														√																										
Self-efficacy	√															√																								
Social support							√																														√			
Surgery history							√																					√												√
Sense of burden																			√																					
Sleep disturbance																							√																	
Social functioning													√																							√				
Social vulnerability																																								√
Shortness of breath																												√												
Schizotypal personality																																				√				
Substance use disorder																			√																					
Satisfaction of treatment																																							√	
Self-reported health status																											√													
Self-rated economic status																								√											√					
Self-perceived disease severity																																						√		
Support by religious community																√																								
Tumor site																																		√				√		√
Traumatic event								√																																
Trouble belching																																					√			
Time since cancer diagnosis							√																	√		√												√	√	
Use of antidepressants																	√																							
Vomiting																												√									√			

### 3.4 Meta-analysis results

Finally, there were 32 risk factors for which data from 2 or more studies could be extracted and analyzed. According to the results of the meta-analysis, suicidal ideation in cancer patients was significantly associated with marital status (OR: 1.73 [1.59–1.88]), living alone (OR: 2.97 [1.80–4.88]), post-traumatic stress disorder (PTSD, OR: 4.00 [2.33–6.87]), panic disorder (OR: 3.68 [1.71–7.90]), education (OR: 2.24 [1.57–3.19]), psychiatric illness history (OR: 5.41 [3.80–7.70]), social functioning (OR: 2.68 [1.58–4.55]), childhood adversity experience (OR: 5.17 [2.88–9.29]), financial problems (OR: 2.42 [1.49–3.92]), pain (OR: 2.10 [1.74–2.52]), depression (continuous data, MD: 6.84 [6.42–7.27]), demoralization (OR: 10.35 [5.29–20.23]) and vomiting (OR: 2.77[2.01–3.82]). All *p*-values (double-tailed) were less than 0.05. In addition to the above factors, there was considerable heterogeneity among the studies (*I*^2^ > 50%) in which the remaining factors were located. Random effects models were employed to estimate the aggregated effect estimates. Preliminary results manifested that residence (OR: 2.79 [1.58–4.91]), depression (count data, OR: 3.99 [2.80–5.69]), anxiety (count data, OR: 3.36 [2.14–5.26]), anxiety (continuous data, MD: 5.43 [4.60–6.26]), chemotherapy (OR: 1.47 [1.07–2.02]), metastasis (OR: 1.69[1.06–2.70]) and the number of physical symptoms (OR: 1.60 [1.15–2.21]) were risk factors for suicidal ideation. All *p*-values (double-tailed) were less than 0.05. A sensitivity analysis was conducted by individually excluding each study, and minimal alterations were observed in the final outcomes pertaining to residential factors, depression (count data), and anxiety. However, after sensitivity analysis, the results of chemotherapy, metastasis and the number of physical symptoms changed significantly, suggesting that the results of pooled analysis were not robust. Detailed meta-analysis results were shown in Table 3.

**Table 3:** Meta-analysis results.

Risk factors	*I*^2^ (%), *P*	Homogeneity/heterogeneity	OR/MD [95%CI], *P*
Marital status (unmarried vs. married)	22, *p* = 0.25	Homogeneity	1.73 [1.59–1.88] ***
Living alone	0, *p* = 0.58	Homogeneity	2.97 [1.80–4.88] ***
Post-traumatic stress disorder	0, *p* = 0.63	Homogeneity	4.00 [2.33–6.87] ***
Panic disorder	0, *p* = 0.78	Homogeneity	3.68 [1.71–7.90] ***
Education (high school and below vs. college and above)	35, *p* = 0.21	Homogeneity	2.24 [1.57–3.19] ***
Psychiatric illness history	0, *p* = 0.46	Homogeneity	5.41 [3.80–7.70] ***
Social functioning (low vs. high)	0, *p* = 0.89	Homogeneity	2.68 [1.58–4.55] ***
Childhood adversity experience	0, *p* = 0.40	Homogeneity	5.17 [2.88–9.29] ***
Financial problems	0, *p* = 0.80	Homogeneity	2.42 [1.49–3.92] ***
Pain (OR)	11, *p* = 0.35	Homogeneity	2.10 [1.74–2.52] ***
Depression (MD)	0, *p* = 0.96	Homogeneity	6.84 [6.42–7.27] ***
Demoralization	50, *p* = 0.16	Homogeneity	10.35 [5.29–20.23] ***
Vomiting	0, *p* = 0.82	Homogeneity	2.77 [2.01–3.82] ***
Residence (rural vs. urban)	67 *	Heterogeneity	2.79 [1.58–4.91] ***
Depression (OR)	97 ***	Heterogeneity	3.99 [2.80–5.69] ***
Anxiety (OR)	76 ***	Heterogeneity	3.36 [2.14–5.26] ***
Anxiety (MD)	82 ***	Heterogeneity	5.43 [4.60–6.26] ***
Chemotherapy	72 *	Heterogeneity	1.47 [1.07–2.02] *
Number of physical symptoms (≥3 vs. <3)	85 ***	Heterogeneity	1.60 [1.15–2.21] **
Pain (MD)	92 ***	Heterogeneity	−0.28 [−2.29–1.73] *p* = 0.78
Religion	90 ***	Heterogeneity	1.61 [0.47–5.51] *p* = 0.45
Gender (female vs. male)	95 ***	Heterogeneity	1.49 [0.96–2.31] *p* = 0.07
Cancer stage (iii and iv vs. i and ii)	96 ***	Heterogeneity	2.17 [0.97–4.87] *p* = 0.06
Smoking	80 **	Heterogeneity	1.36 [0.83–2.24] *p* = 0.22
Employment	84 ***	Heterogeneity	1.15 [0.69–1.94] *p* = 0.59
Age (MD)	88 ***	Heterogeneity	−0.80 [−3.46–1.85] *p* = 0.55
Recurrence	82 **	Heterogeneity	1.18 [0.61–2.26] *p* = 0.63
Hopelessness (MD)	84 **	Heterogeneity	1.88 [−0.06–3.81] *p* = 0.06
Performance status (MD)	92 ***	Heterogeneity	−5.14 [−10.94–0.67] *p* = 0.08
Bipolar/schizophrenia	97 ***	Heterogeneity	3.00 [0.35–25.86] *p* = 0.32
Metastasis	92 ***	Heterogeneity	1.69 [1.06–2.70] *
Surgery history	100 ***	Heterogeneity	4. [0.09–236.08] *p* = 0.44

*p < 0.05, **p < 0.01, ***p < 0.001.

Specific information on forest plots of risk factors and funnel plots of published bias were provided in Appendices 1, 2. For depression, funnel plot of 17 included studies showed partial asymmetry, suggesting possible publication bias.

## 4 Discussion

This systematic review and meta-analysis is the first comprehensive summary of risk factors associated with suicidal ideation among cancer patients, to our knowledge. Forty studies from 13 countries were included, with sample sizes ranging from 60 to 382,266. Among them, there are 30 studies were cross-sectional design, 9 for cohort, 1 for case–control study. The Quality of the literature was assessed by the Newcastle-Ottawa scale and the Agency for Healthcare Research and Quality. The quality of the included studies were moderate or above, and they met the requirements with high reliability.

We identified a large number of studies reporting nearly 122 risk factors associated with suicidal ideation, synthesizing studies on influencing factors over the last 23 years. The review findings indicate a prevalence rate of suicidal ideation ranging from 0.27 to 53.3% among cancer patients. Furthermore, suicidal ideation in cancer patients was significantly associated with depression, marital status, living alone, post-traumatic stress disorder, panic disorder, education, psychiatric illness history, social functioning, childhood adversity experience, financial problems, pain, residence, anxiety, vomiting and demoralization.

Our review demonstrated that unmarried, less educated, living alone, living in rural, having lower quality of social functioning, and having financial problems were linked to suicidal ideation. The findings of Erryk S. Katayama et al. illustrated a consistent association between unmarried status and an increased risk of suicidal ideation among cancer patients, aligning with our own results (Katayama et al., 2023). Physically, spouses exhibit heightened awareness of patients’ needs, while emotionally, they demonstrate greater understanding of patients’ preferences. In addition, the presence and support of a spouse can foster treatment adherence among married patients, thereby enhancing the overall treatment trajectory and ultimately improving outcomes (Liao et al., 2019; Luo et al., 2022). Isolation seems to be distinctly prevalent among patients who live alone, and lack of family support may lead to isolation and suicidal thoughts (Henry et al., 2018). The association between lower quality social functioning and suicidal ideation may be attributed to cancer patients’ increasing social disengagement during their prolonged hospital stays, and it can lead to a perception that their abilities are underutilized and hindered in achieving their social values (Jung and Yun, 2022). In hospital, proper organization of group activities such as group exercises and singing competitions can enhance the connection between cancer patients and society. And in community, it is imperative to engage in frequent visitations and communication, or organize community activities aimed at enhancing their social functioning, maintaining social roles, and fostering a sense of worthiness. Patients with higher levels of education or living in urban were more involved in treatment decisions, had more access to disease information, and had adequate communication with their physicians (Tang et al., 2022). On the contrary, in low educated patients or patients living in rural, treatment decisions were mostly made by family members, and even some of these patients did not have a complete understanding of their condition. Therefore, they might be higher possibility to get suicidal ideation. It is crucial for cancer patients to promptly acquire comprehensive knowledge related to their condition, enabling them to gain a better understanding of the disease. Simultaneously, providing care and encouragement to cancer survivors is essential in empowering them to confront cancer with greater resilience. Patients facing financial difficulties are particularly concerned about imposing burdens on their families or incurring substantial expenses without experiencing improvement, which can contribute to suicidal ideation (Wilson et al., 2005). Medical professionals and social workers can seek assistance based on the patient’s condition, for instance, organizing patient donations or coordinating with social welfare organizations to raise funds.

In addition, we noted that childhood adversity experience, depression, anxiety, demoralization, post-traumatic stress disorder and panic disorder were risk indicators of suicidal ideation. There is a plethora of evidence that childhood adversity experience is significantly associated with suicidal ideation (Henry et al., 2018; Zhang et al., 2020). Specific types of childhood traumas, namely severe emotional neglect and emotional violence, can trigger the onset of social isolation in an individual’s psychological well-being. Consequently, individuals in this situation may come to believe that they lack connection and a sense of belonging, which may increase their vulnerability to suicidal tendencies (Zhang et al., 2020). The same goes for post-traumatic stress disorder and panic disorder. Stressful traumatic experiences may drain patients’ mental energy and resources to a large extent, which makes them more susceptible to mental disorders (Mellon et al., 2007). Demoralization may have a strong negative impact on the psychological state of cancer patients. When patients are unable to effectively manage stress, they may feel helpless and hopeless, which leads to a loss of meaning and purpose in life (Xu et al., 2019). Some studies have demonstrated that music intervention exerts a substantial positive influence on anxiety, depression, pain, fatigue, and overall quality of life (Bulotiene and Pociute, 2019). The hospital can offer music intervention and psychological lectures to cancer patients, aiming to facilitate the elimination of negative emotions and alleviate emotional exhaustion. Meanwhile, their family members should provide unconditional positive attention and ample familial support.

What’s more, anxiety and depression are the predominant risk factors for suicidal ideation among cancer patients. Anxiety, through the alteration of brain chemistry, particularly affecting serotonin, plays a significant role in mood regulation, thereby increasing the risk of suicidal ideation (Bachmann, 2018). As patients endure long-term treatment and the adverse reactions brought by treatment, anxiety is likely to turn into depression (Luo et al., 2022). Cancer patients with depression are much easier to experience suicidal ideation than other patients, because depression is an independent cause of suicidal ideation regardless of the cancer (Stanbouly et al., 2023). Cancer patients could generate high levels of psychological distress resulting from cancer diagnosis, treatment and social isolation (Abdel-Rahman, 2019). Moreover, individuals with depressive symptoms, including hopelessness, insomnia, decreased appetite, social isolation, fatigue, and diminished self-worth, are significantly more vulnerable to experiencing suicidal ideation. It is widely recognized that depression among cancer survivors is frequently overlooked or underestimated in the context of cancer care (Passik et al., 1998). Once the depressed patients through various channels reveal negative, pessimistic mood; recently suffered a serious event difficult to remedy; recently had strong hostility and aggression and treatment of the attitude of the abnormal behavior needs medical staff to cause enough attention, because it is likely to be an important signal of suicide. In conclusion, the early identification and intervention of depression among cancer survivors holds significant potential as a pivotal strategy in preventing suicide in this population (Balcı Şengül et al., 2014; Akechi, 2020). The findings of various studies have demonstrated that mindfulness meditation can offer solace and enhance self-regulation among cancer patients, significantly bolstering their psychological resilience and safeguarding them against impulsive decision-making. Consequently, individuals undergoing this practice develop a comprehensive comprehension and endurance, thereby mitigating the impact of cancer on their well-being (Panta, 2018). As a result, healthcare professionals are encouraged to appropriately guide patients in engaging with mindfulness meditation as a means to alleviate their psychological distress.

Cancer patients with vomiting, pain and psychiatric illness history were noted growing trend of suicidal ideation. They are greatly affected by a range of symptoms that come with treatment. The treatment cycle is long, the cost is high, and the side effects are obvious, such as vomiting, changes in appearance and sleep disorders (Choi et al., 2014). Vomiting may cause the patient to feel a loss of appetite, resulting in insufficient daily energy intake to cope with daily life and treatment, which may fall into a vicious cycle, loss of confidence in life and even suicidal ideation (Tang et al., 2022). Cancer patients who experience no or mild pain tend to have reduced psychological distress and an enhanced quality of life. Conversely, individuals dealing with severe pain might prematurely halt coping efforts, contemplate escape strategies from the pain, and even contemplate suicide (Suarez-Almazor et al., 1997). Furthermore, research has indicated that patients are at particularly high risk of severe emotional and physical distress when disclosing a cancer diagnosis and treatment (Anguiano et al., 2012). For cancer patients with a history of mental illness, they face higher levels of psychological stress than the general population (Hyer et al., 2021). Mental deterioration may impair a patient’s ability to cope with cancer and even lead to suicide. Therefore, appropriate psychological interventions for patients with both a cancer diagnosis and a mental disorder are essential to reduce suicide risk. At the same time, in clinical work, more and timely attention should also be paid to cancer survivors with pain and physical discomfort symptoms to eliminate suicidal ideation.

Despite the positive effects of psychological interventions on alleviating negative emotions and suicidal ideation in cancer patients, cancer patients may lack motivation to participate in psychological intervention programs (Sebri et al., 2022). In the future, strategies to enhance the motivation of cancer patients to participate in psychological intervention can be explored. The personalized design of psychological intervention and the combination of psychological intervention with other means (such as musical intervention or social support) can further enhance the motivation of cancer patients to participate in psychological intervention programs, thereby reducing the suicidal ideation of patients.

## 5 Limitations

There are several limitations of studies included in our review. First, the included studies were all written in English, which limits knowledge sharing among large-scale researchers writing in other languages. Further, a large number of moderate quality articles were included in the study, and it is suggested that more high-quality and large sample original studies should be included to supplement and verify it in the future. Additionally, considering its limited reliability and lack of academic control, we opted not to include the gray literature in our study. However, we acknowledge its potential value and anticipate future research in this area. Finally, there were few literatures involving panic disorder and living alone in the included factors, which may lead to a certain bias in the Meta-analysis and affect the results of the Meta-analysis. Future studies in this field are needed to explore the relationship between them and suicidal ideation, contributing to the robustness of the conclusions drawn from our meta-analysis. Furthermore, further empirical research is necessary to validate certain risk factors for suicidal ideation among cancer patients that are not well documented in current existing literature.

## 6 Conclusion

Compared with cancer patients without suicidal ideation, depression, marital status, living alone, post-traumatic stress disorder, panic disorder, demoralization, vomiting, education, psychiatric illness history, social functioning, childhood adversity experience, financial problems, pain, residence and anxiety were identified as major risk factors for having suicidal ideation. Our review has the potential to assist healthcare providers in early identification of cancer survivors who are at a high risk of suicidal ideation, enabling them to proactively implement suicide prevention interventions.

## Data availability statement

The original contributions presented in the study are included in the article/Supplementary material, further inquiries can be directed to the corresponding author.

## Author contributions

JC: Formal analysis, Methodology, Writing – original draft. ZP: Formal analysis, Methodology, Writing – original draft. DH: Project administration, Supervision, Writing – review & editing. JW: Data curation, Writing – review & editing. YL: Project administration, Supervision, Writing – review & editing.
