# Supplementary material for: Risk factors associated with suicidal ideation among cancer patients: a systematic review and meta-analysis
Source: Front Psychol. 2024 Jan 8;14:1287290. doi: 10.3389/fpsyg.2023.1287290 (PMC10800839; doi:10.3389/fpsyg.2023.1287290)
Supplement: Supplementary file 1 [file Table_1.DOCX]

**Supplementary Appendix-1:** Forest plots of risk factors

**
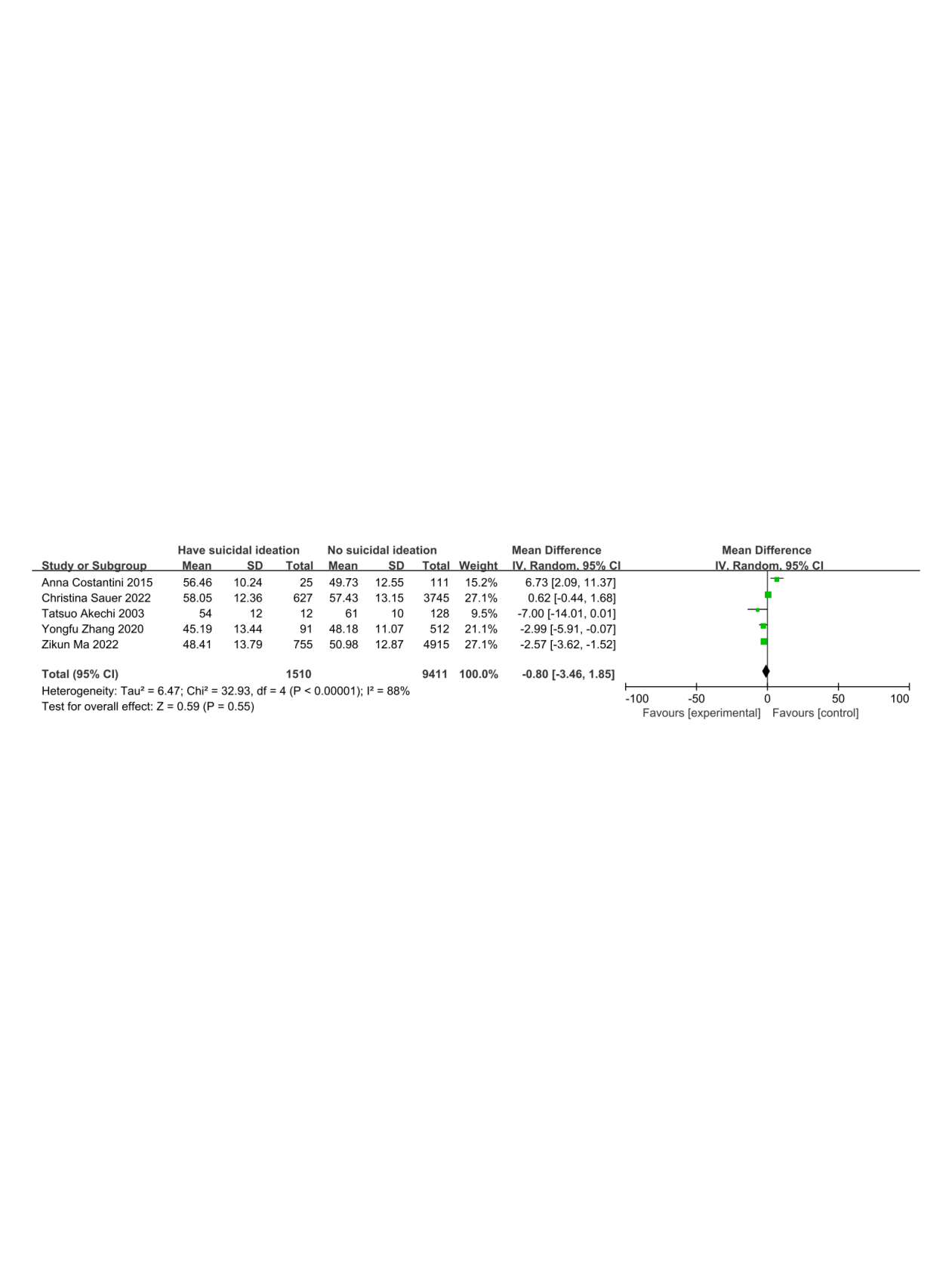
**

Appendix-1 Age (MD)

**
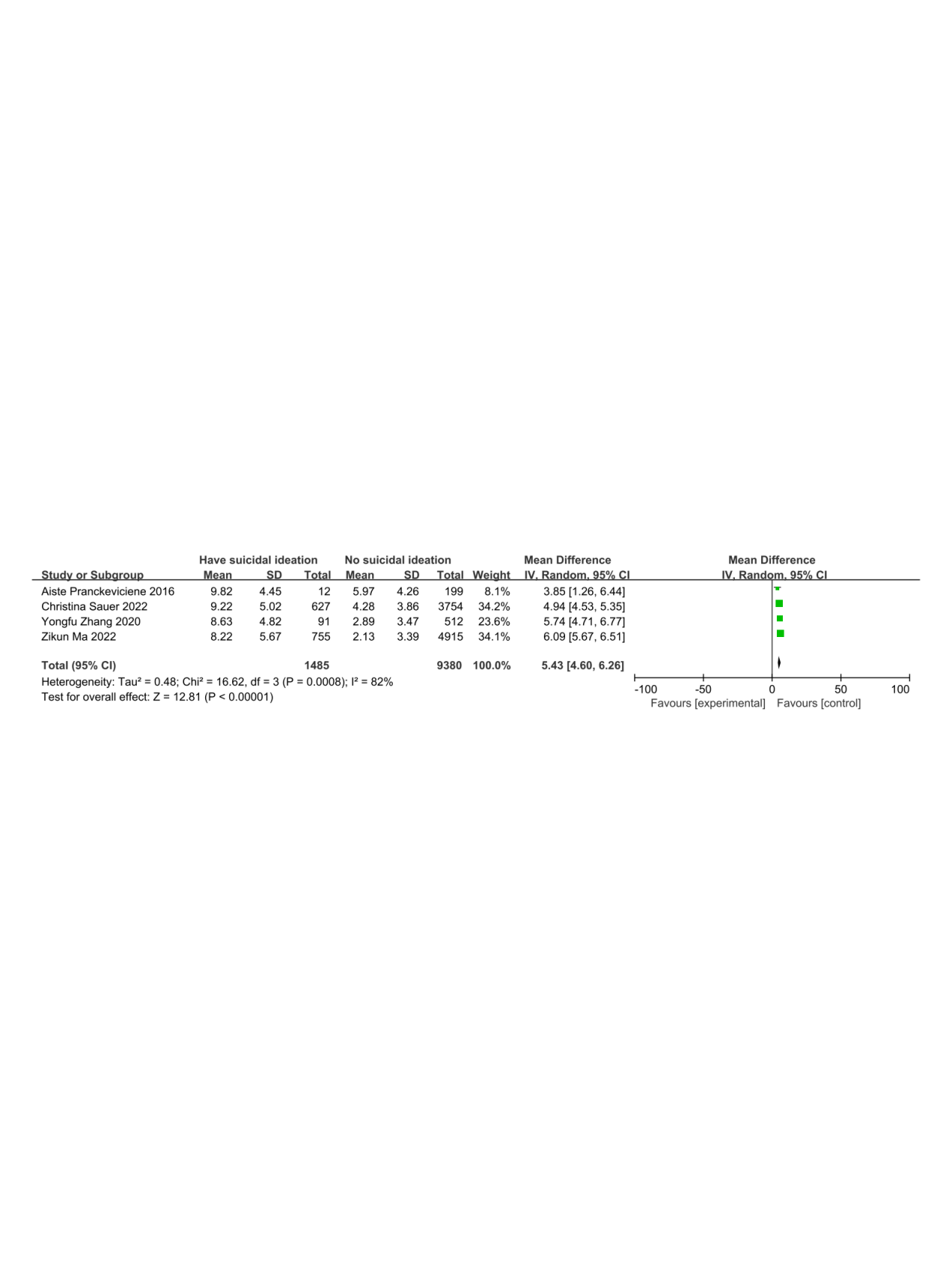
**

Appendix-1 Anxiety (MD)

**
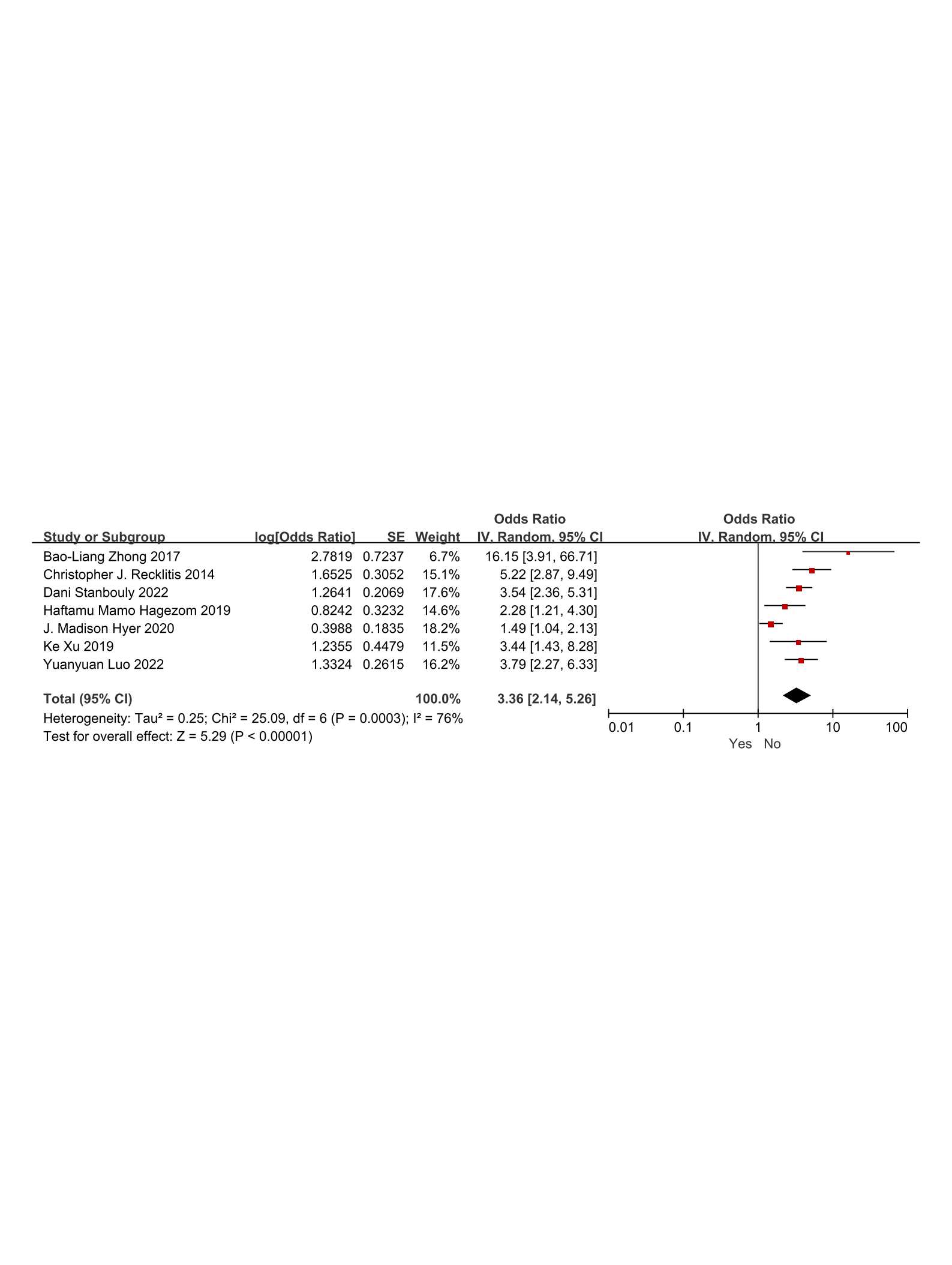
**

Appendix-1 Anxiety (OR)

**
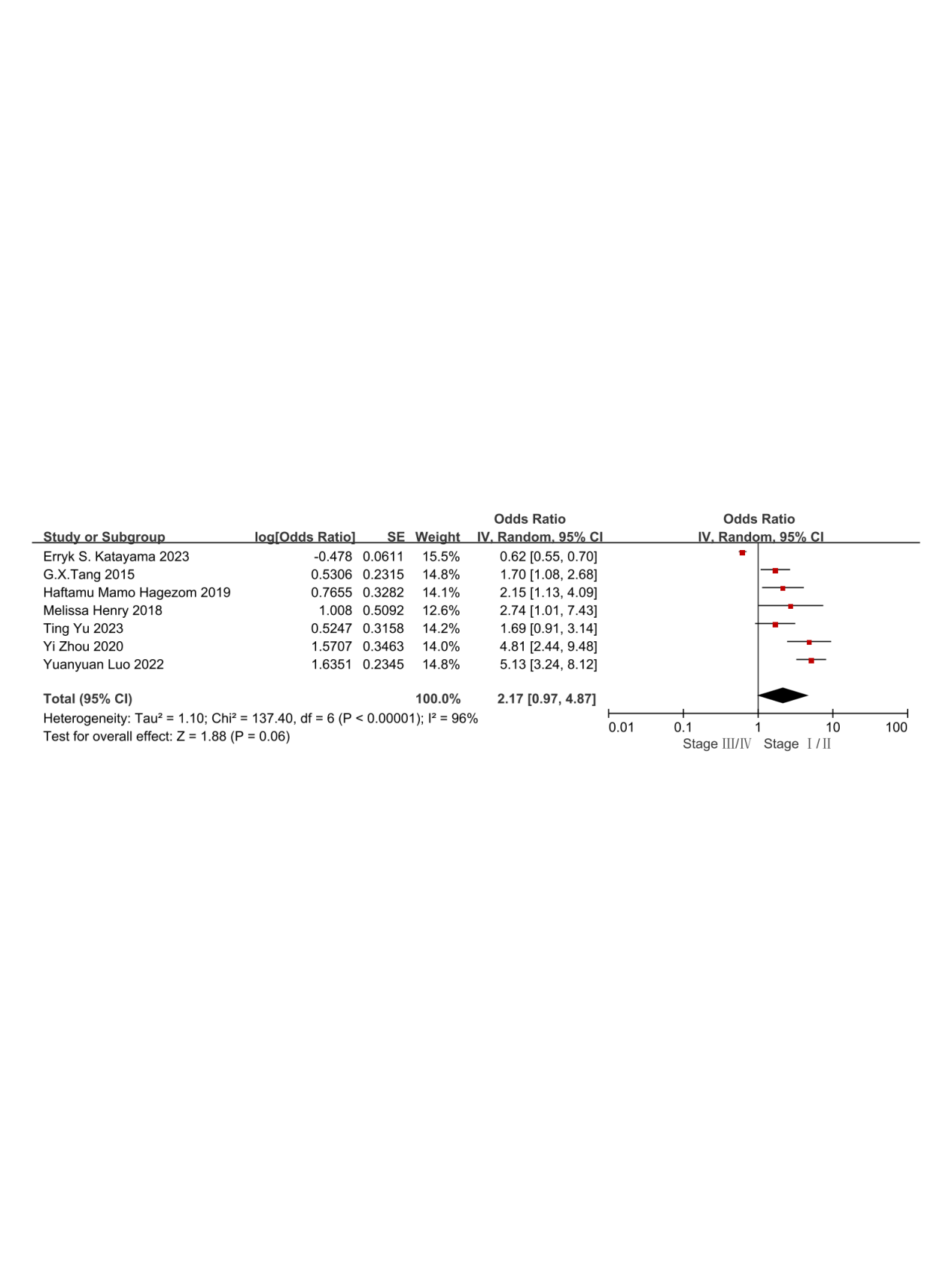
**

Appendix-1 Cancer stage (Stage Ⅲ/Ⅳ vs. Stage Ⅰ/Ⅱ)

**
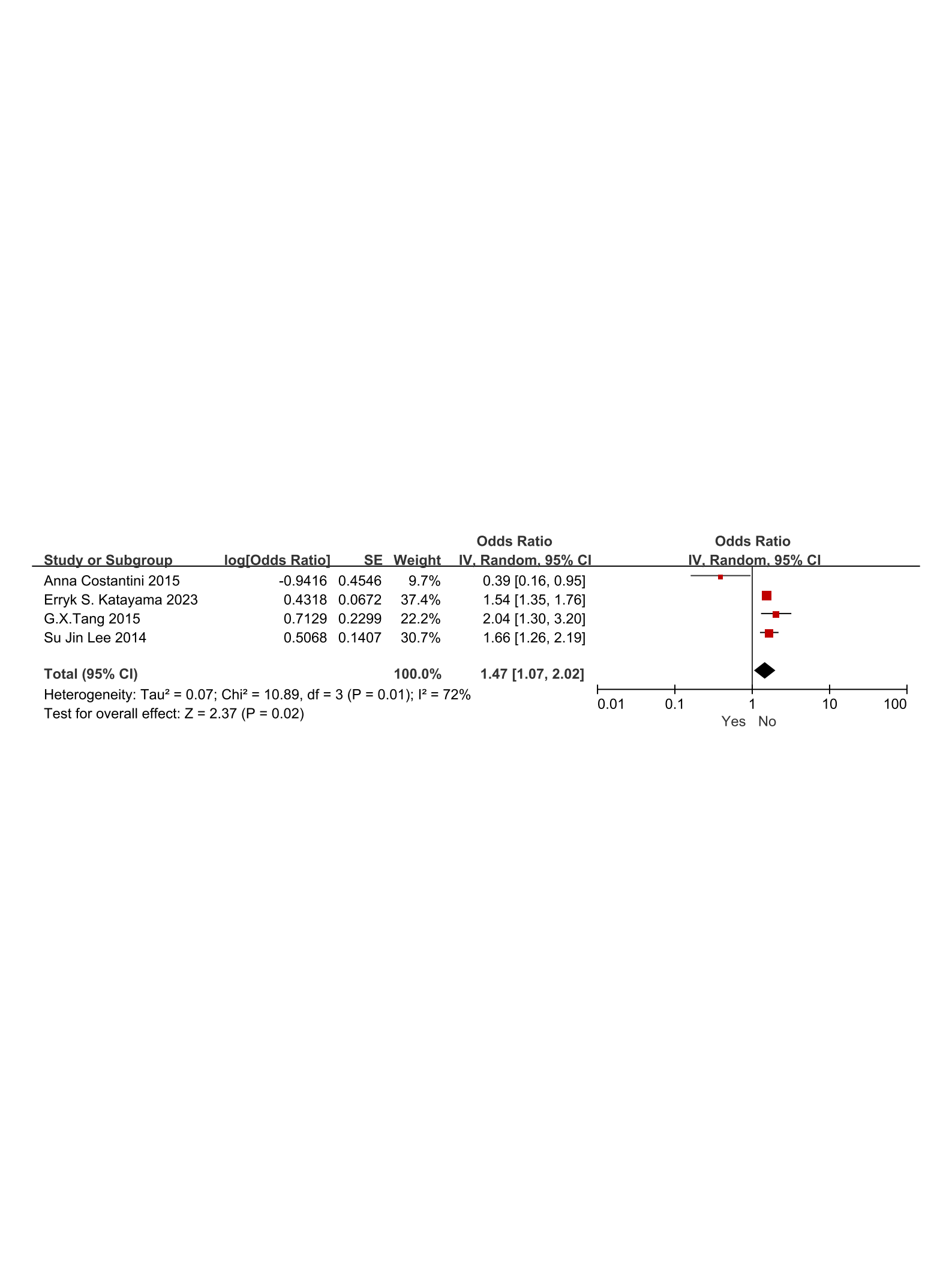
**

Appendix-1 Chemotherapy history

**
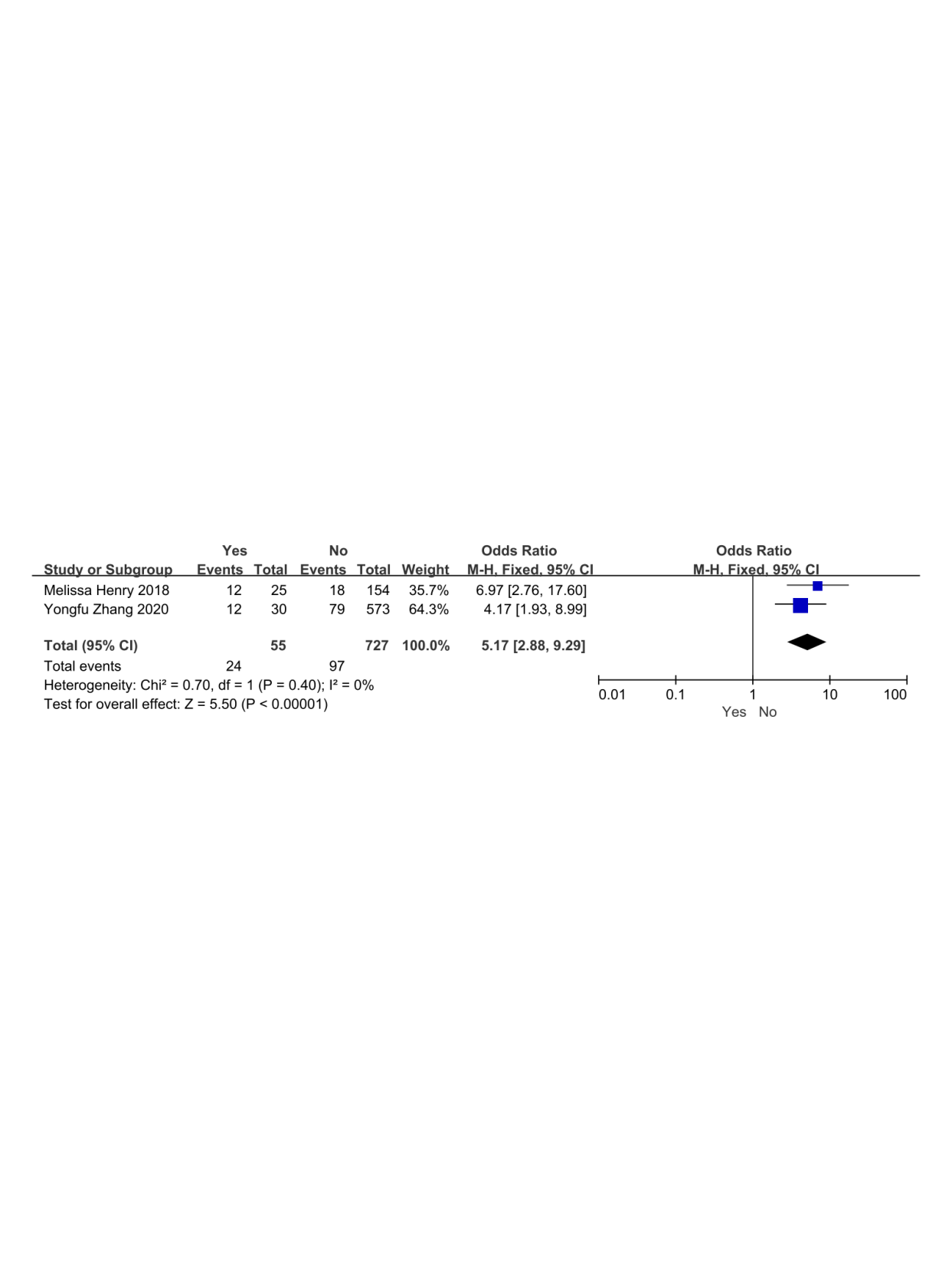
**

Appendix-1 Childhood adversity experience

**
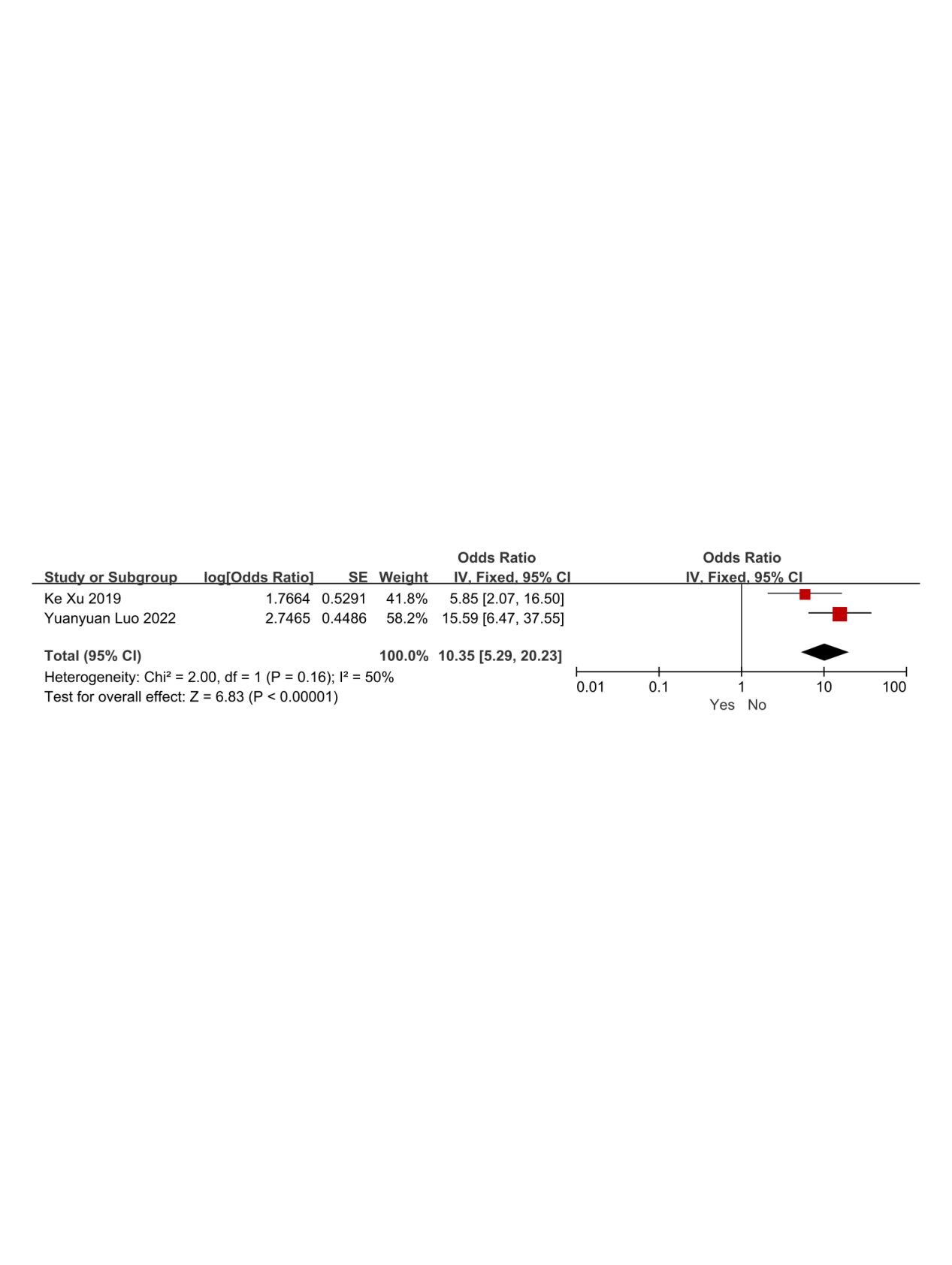
**

Appendix-1 Demoralization

**
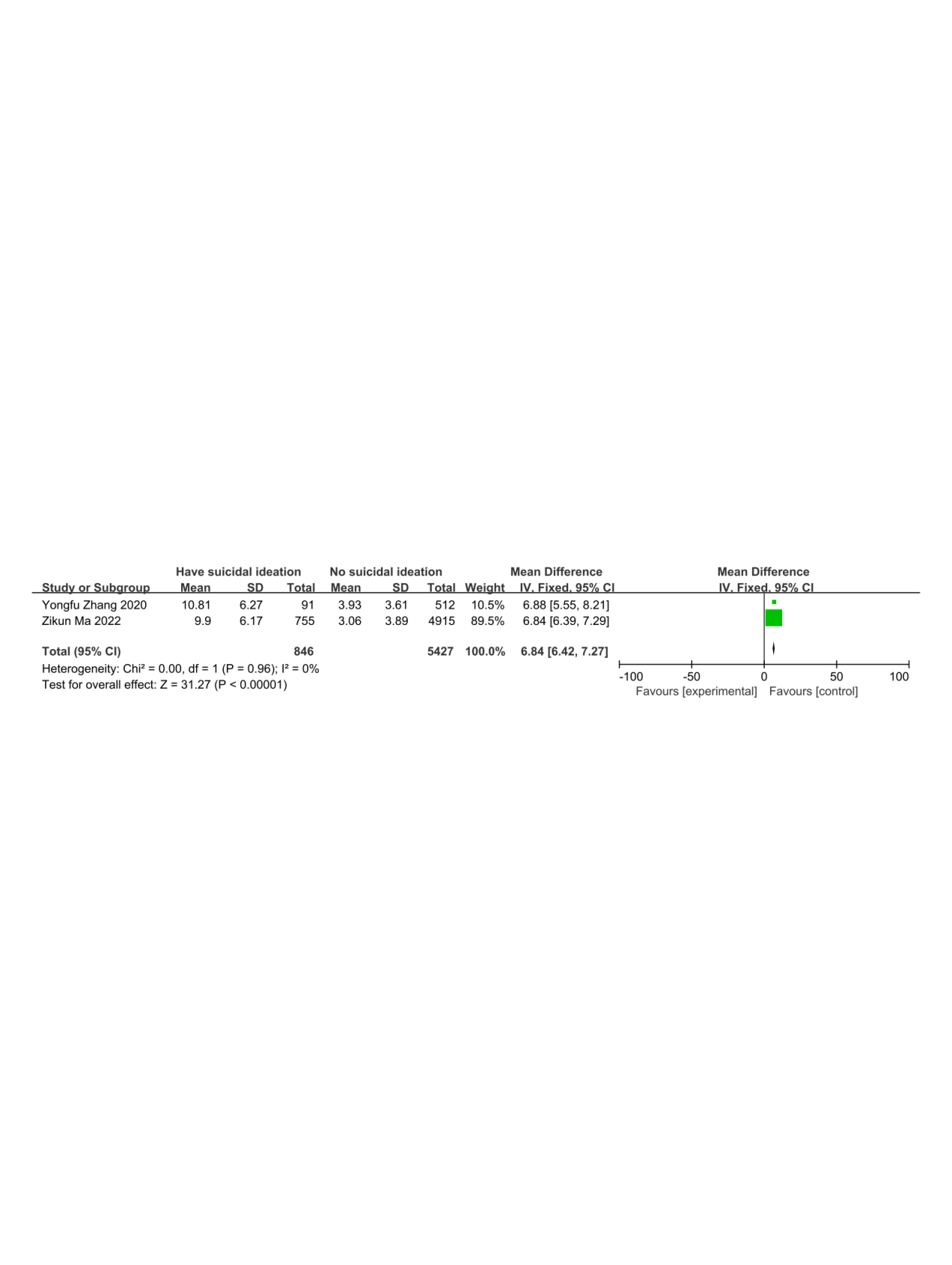
**

Appendix-1 Depression (MD)

**
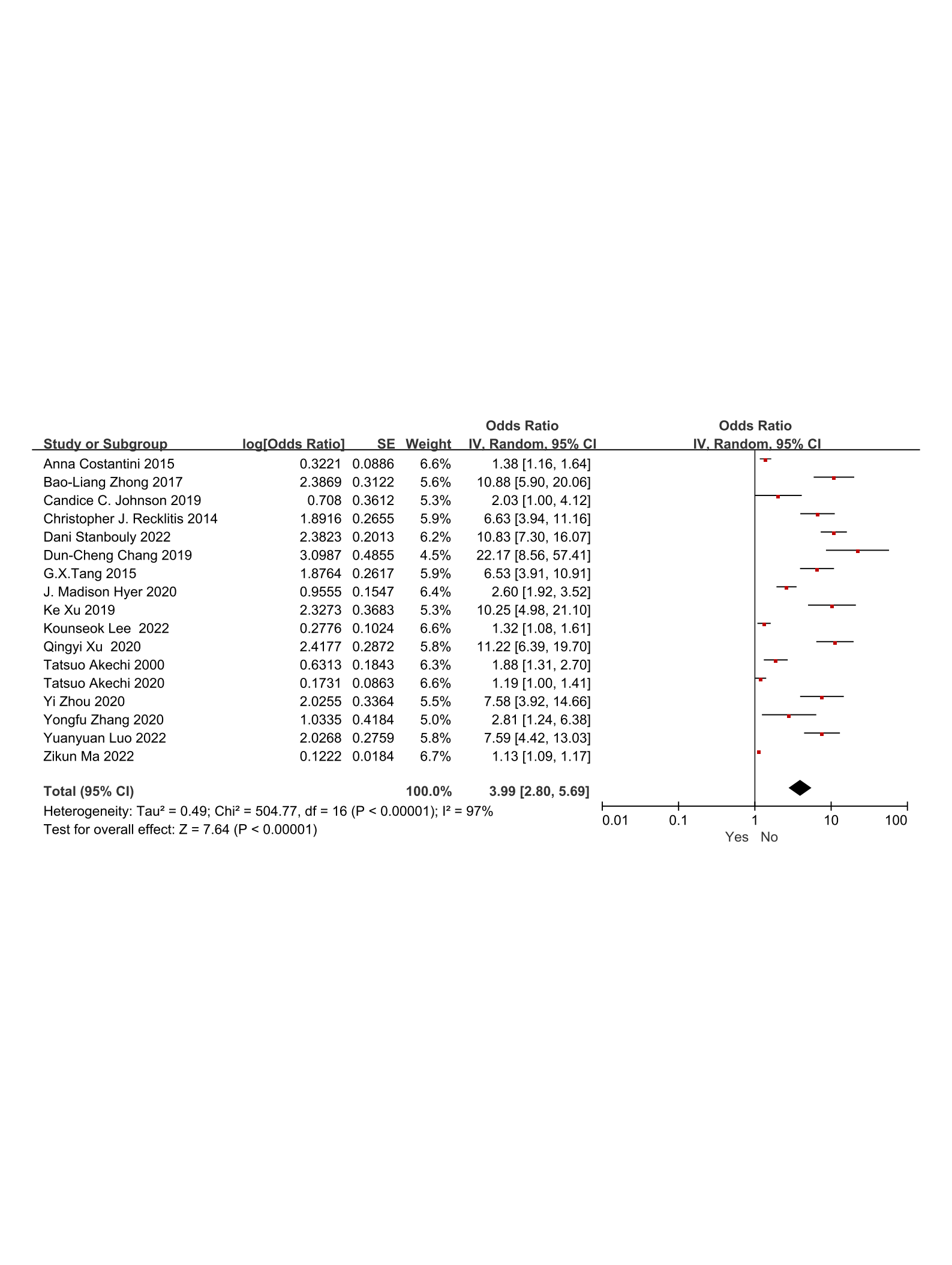
**

Appendix-1 Depression (OR)

**
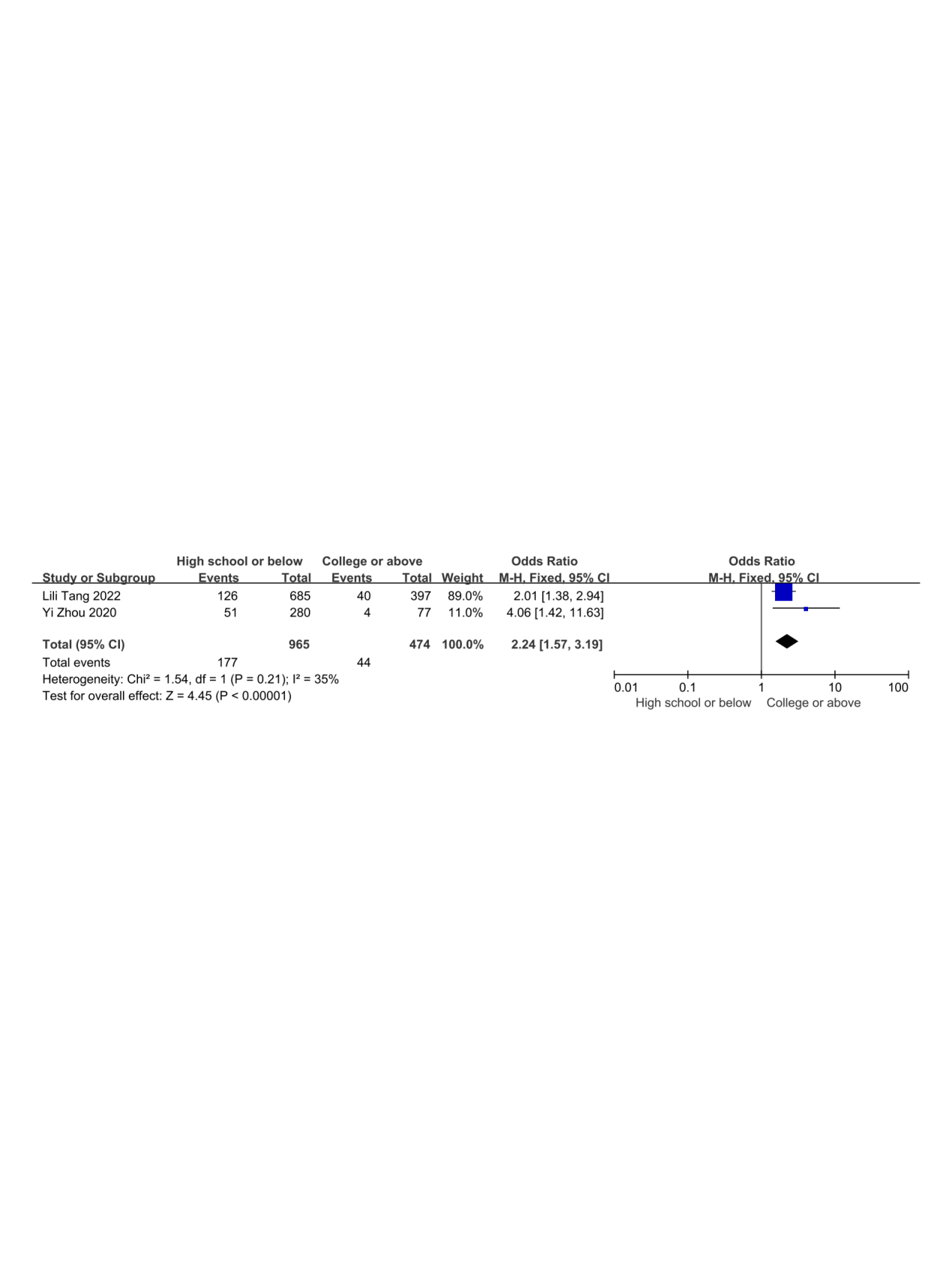
**

Appendix-1 Education (high school and below vs. college and above)

**
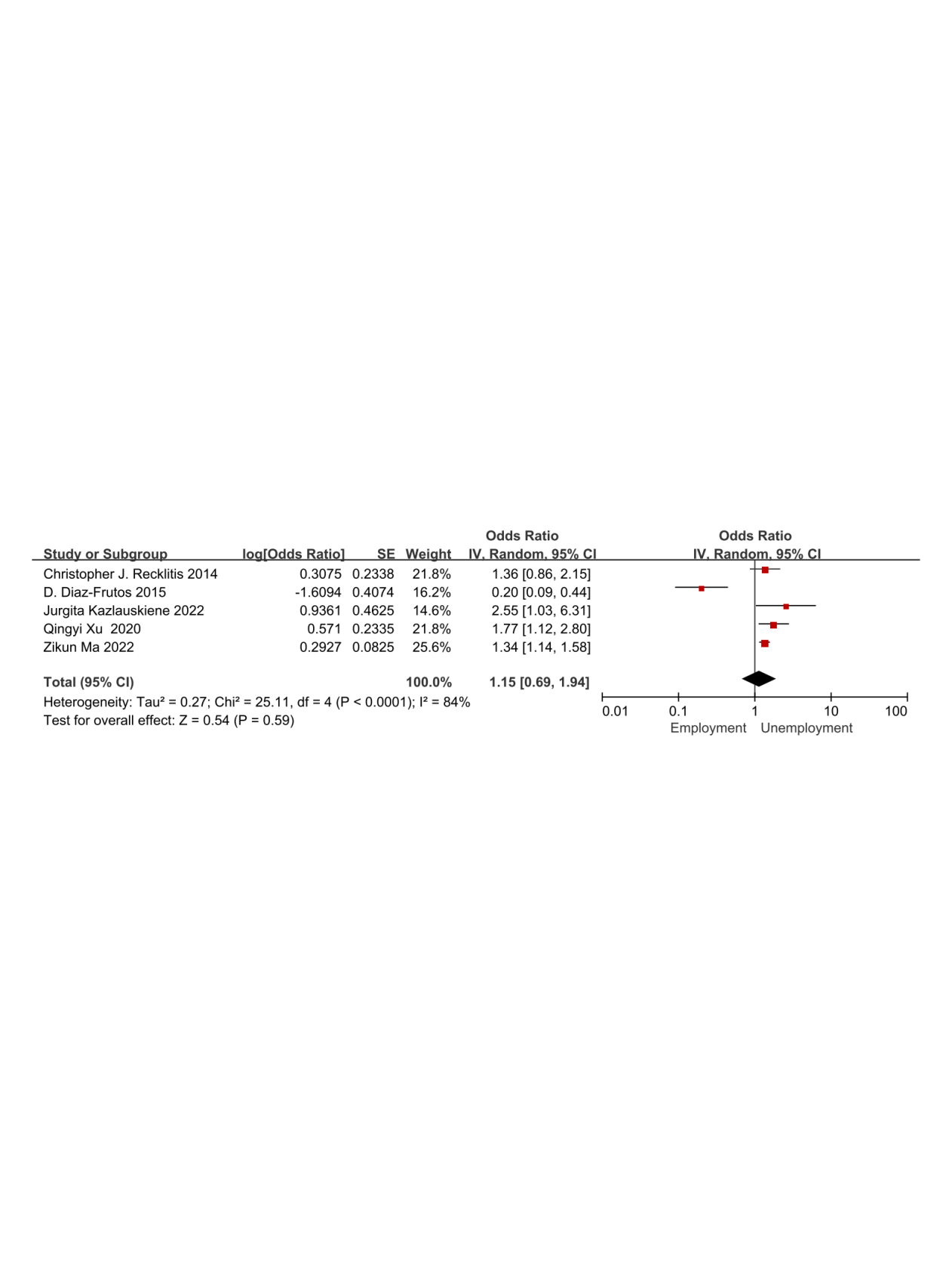
**

Appendix-1 Employment

**
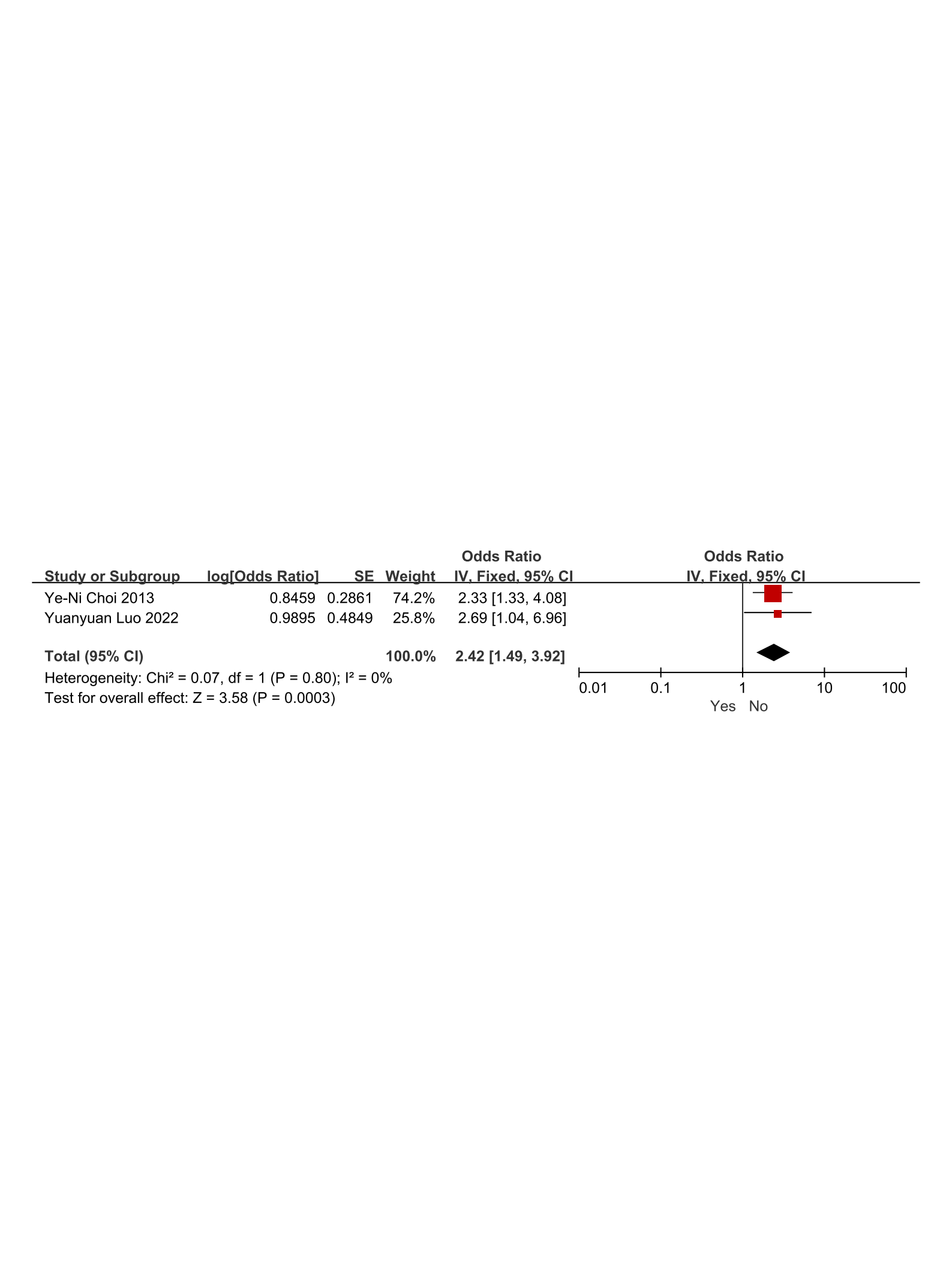
**

Appendix-1 Financial problems

**
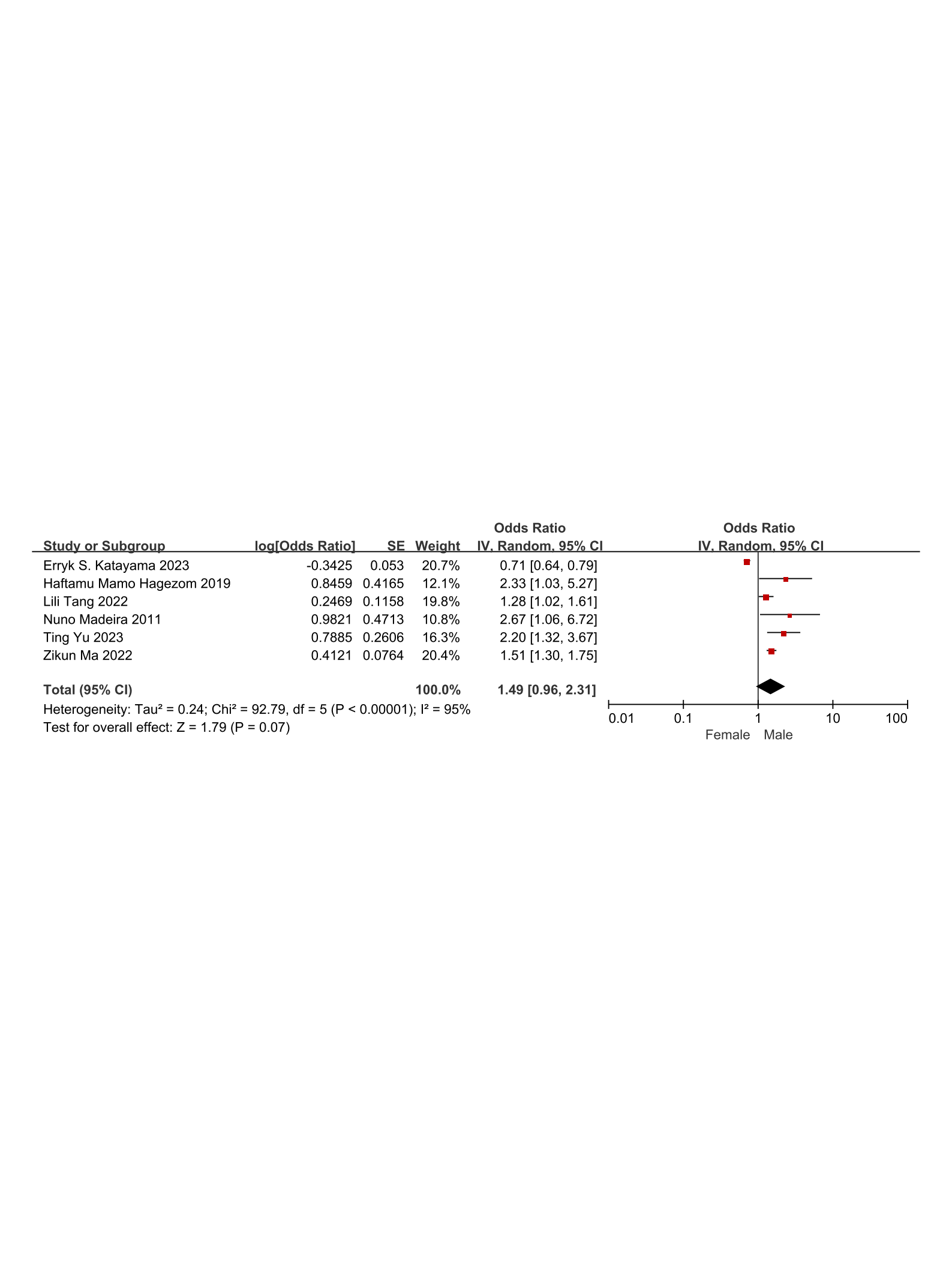
**

Appendix-1 Gender (female vs. male)

**
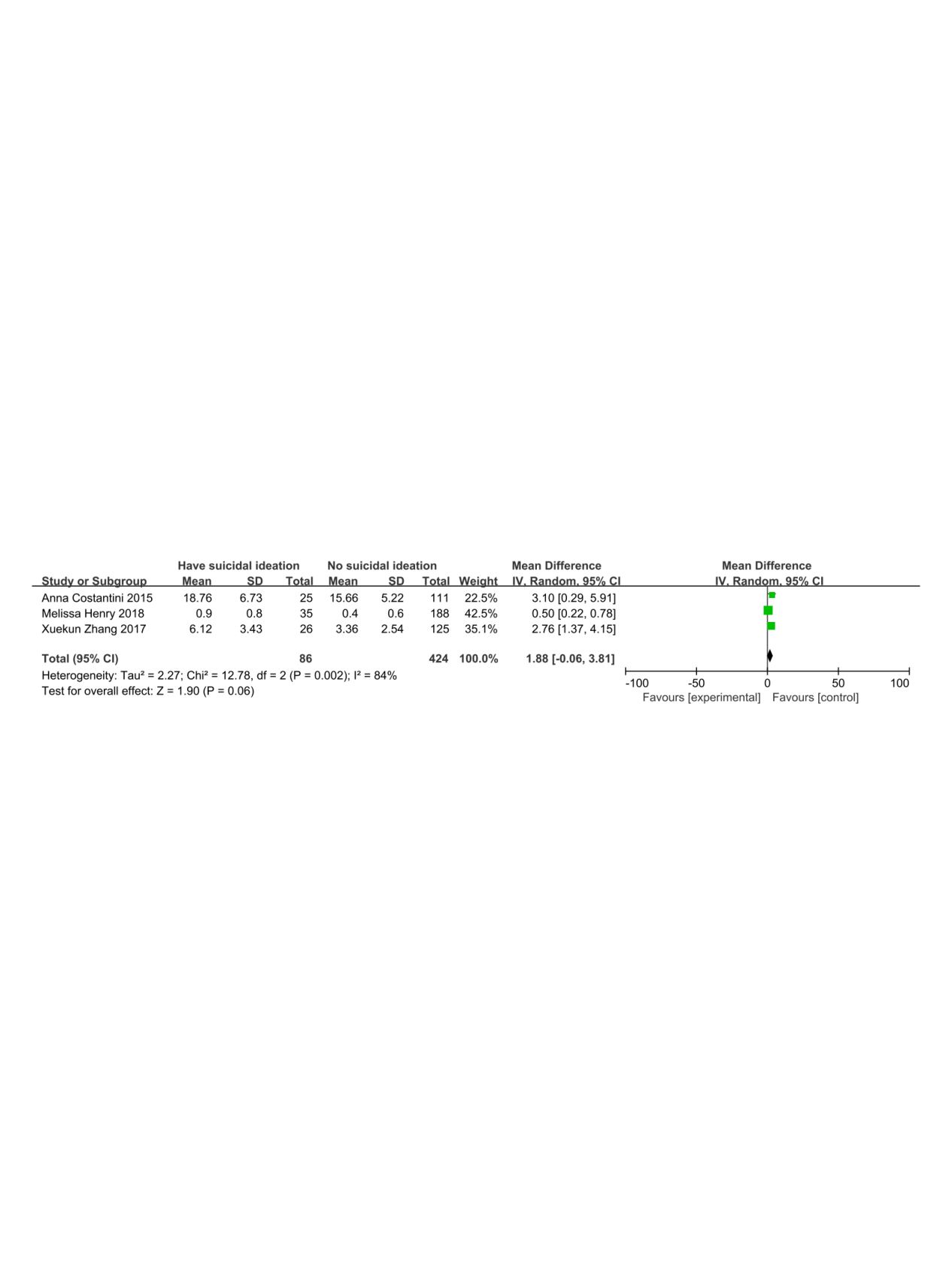
**

Appendix-1 Hopelessness

**
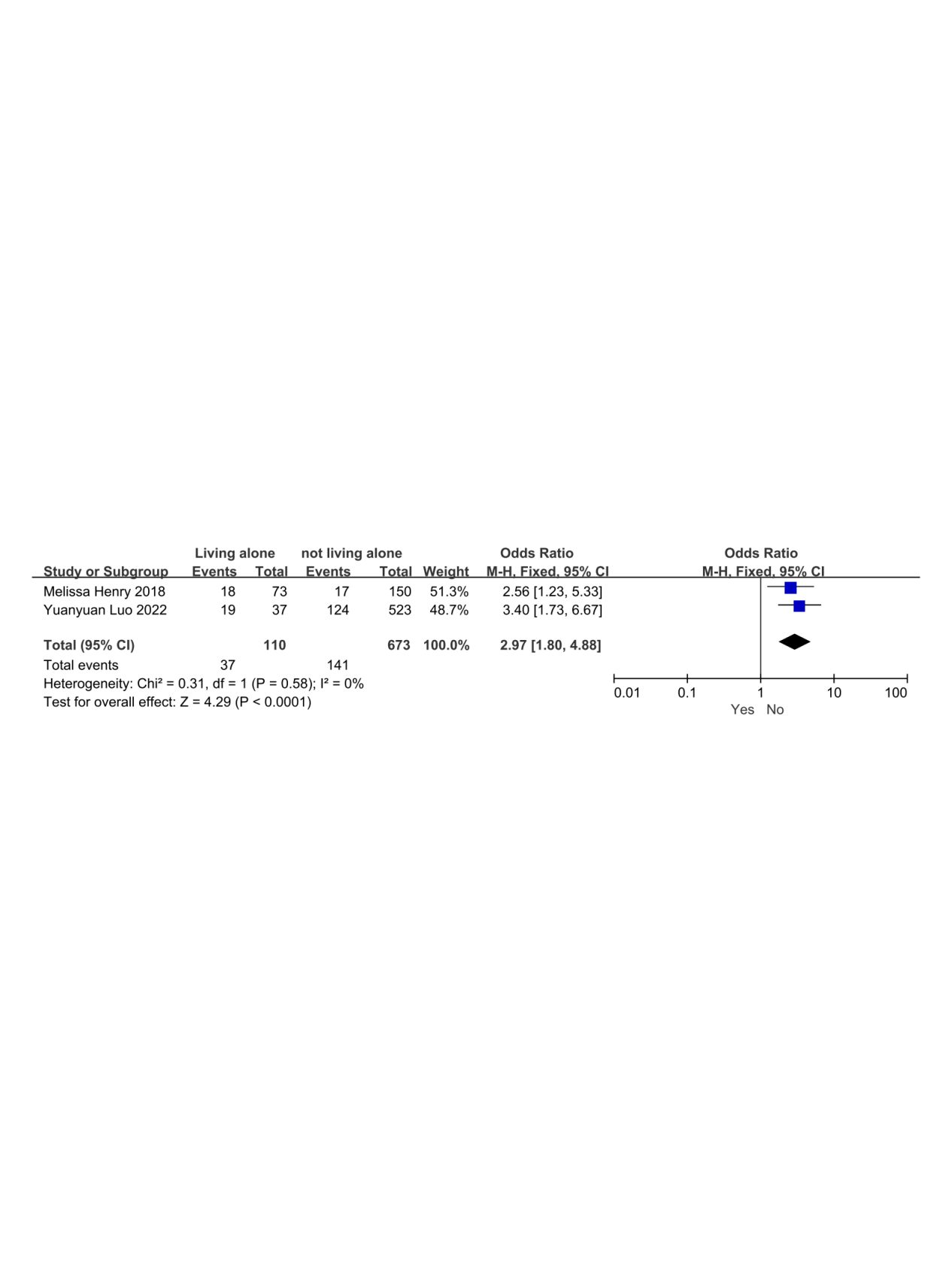
**

Appendix-1 Living alone

**
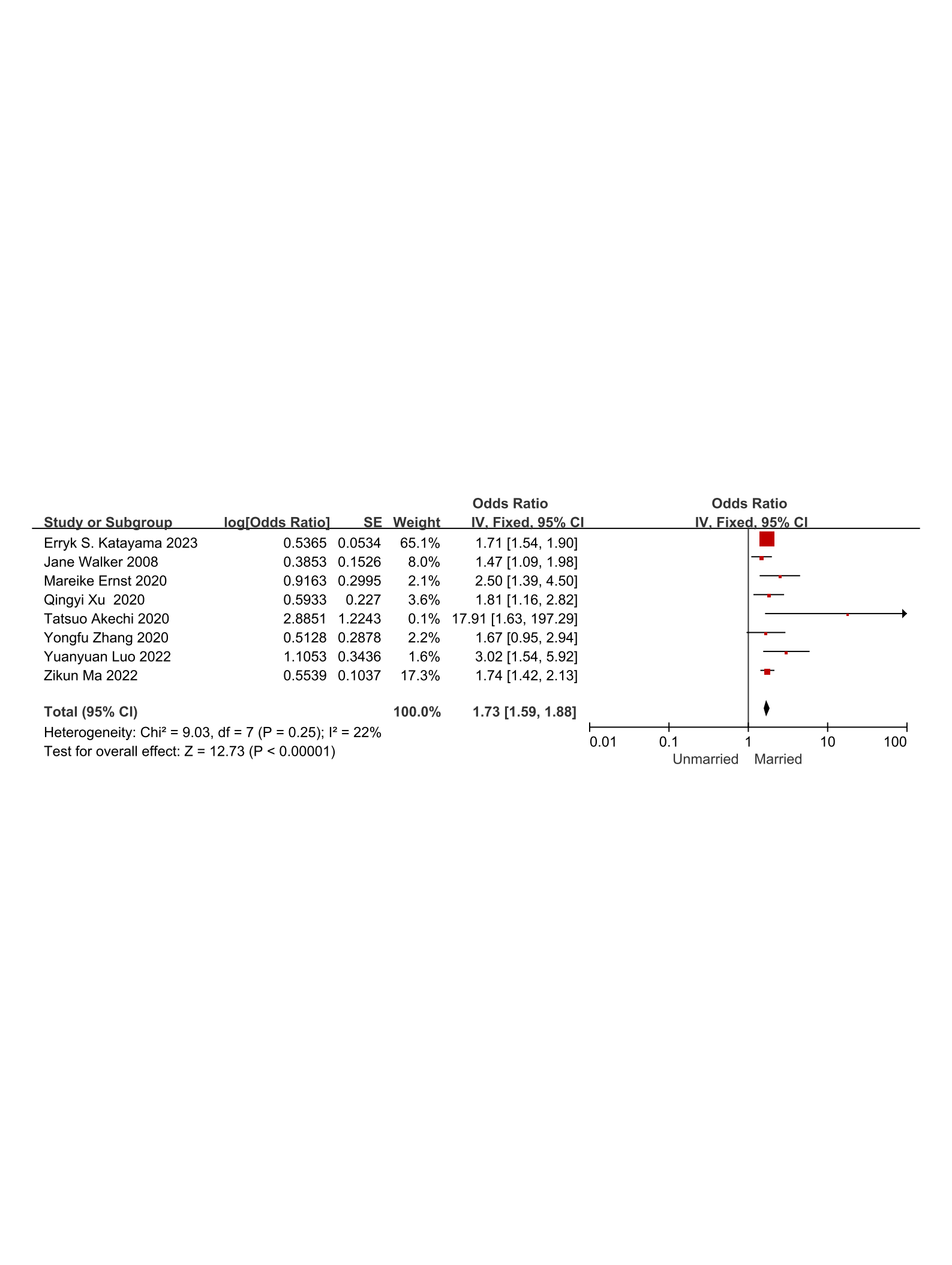
**

Appendix-1 Marital status (unmarried vs. married)

**
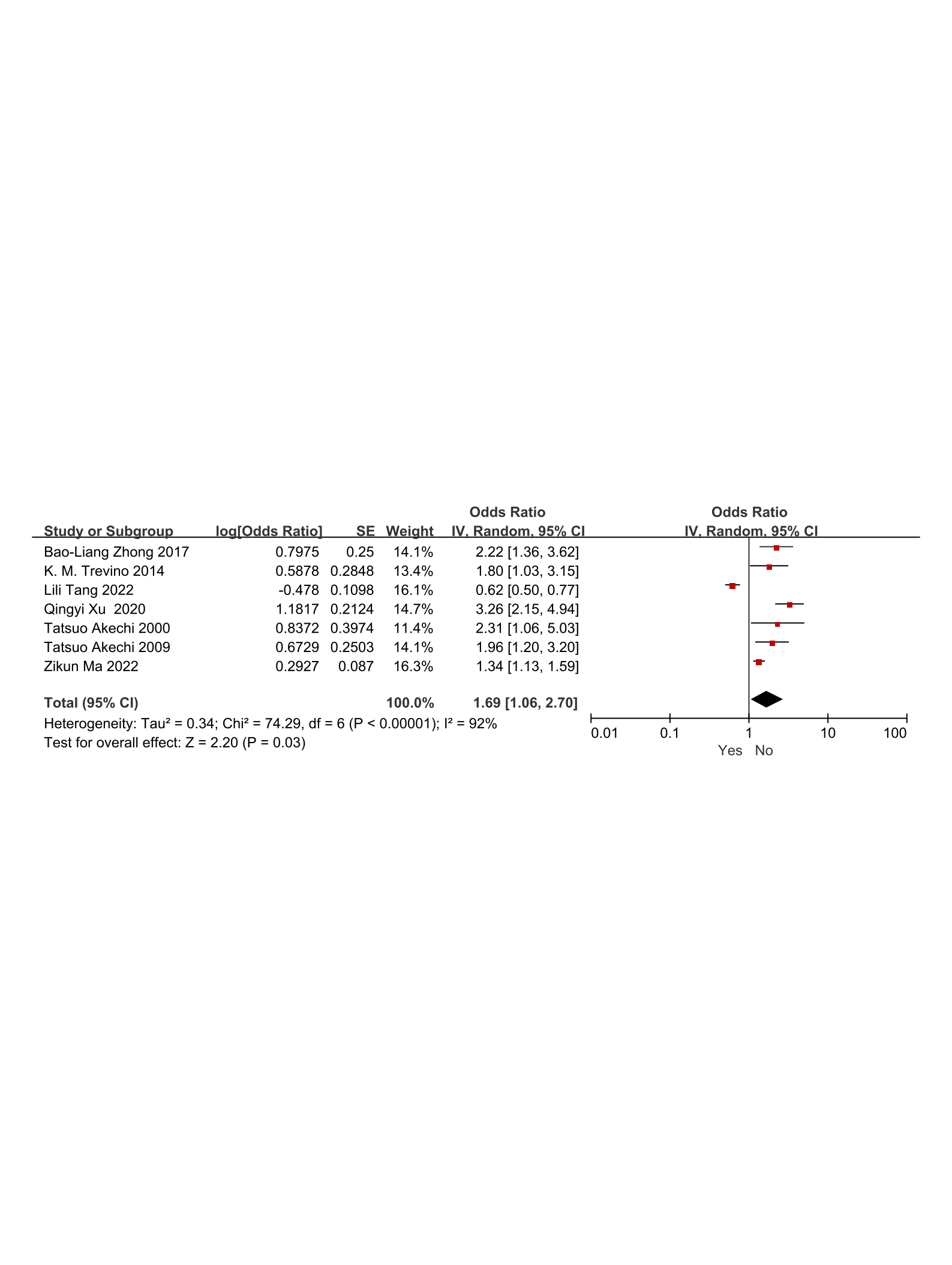
**

Appendix-1 Metastasis

**
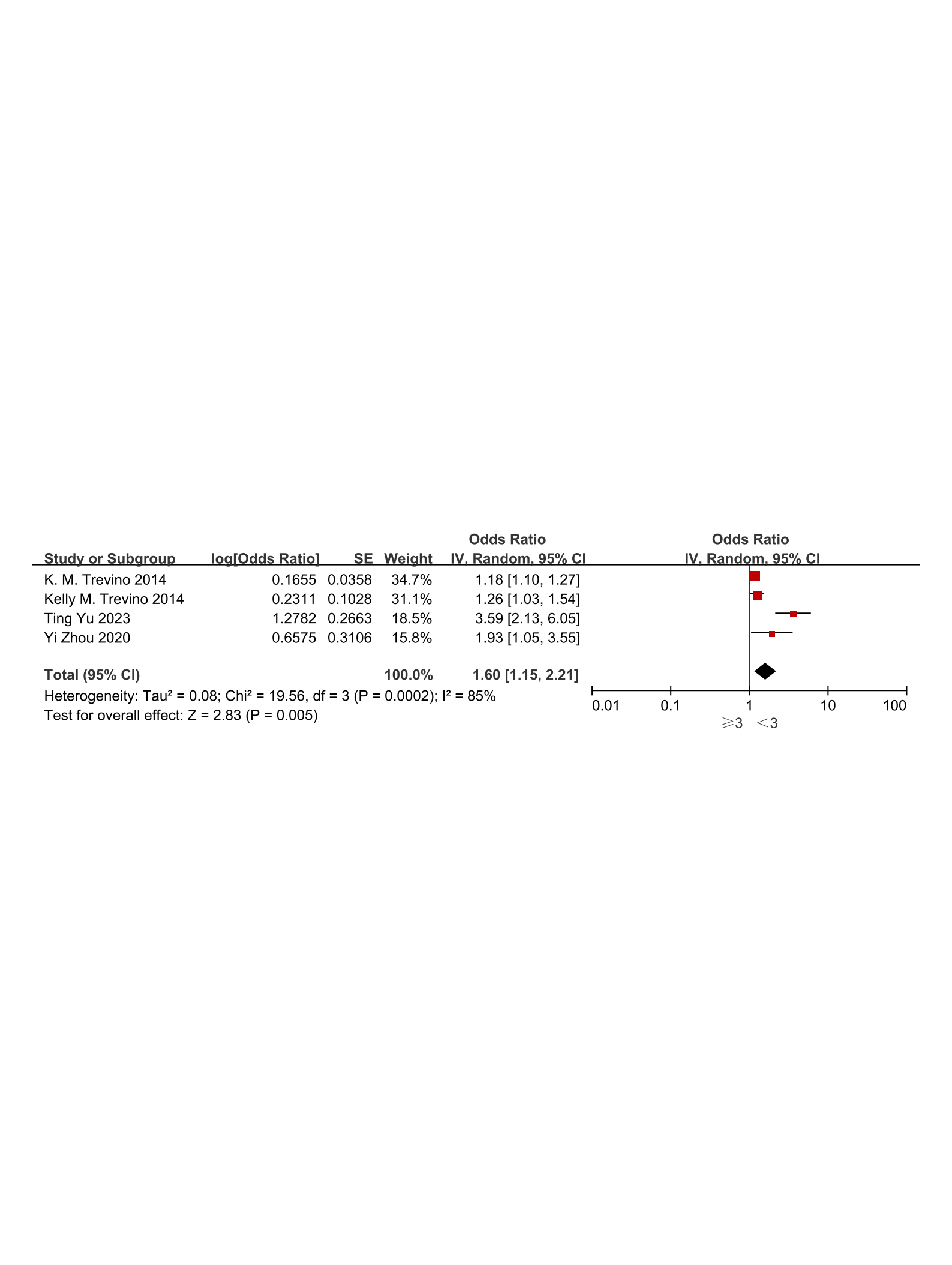
**

Appendix-1 Number of physical symptoms (≥3 vs. ＜3)

**
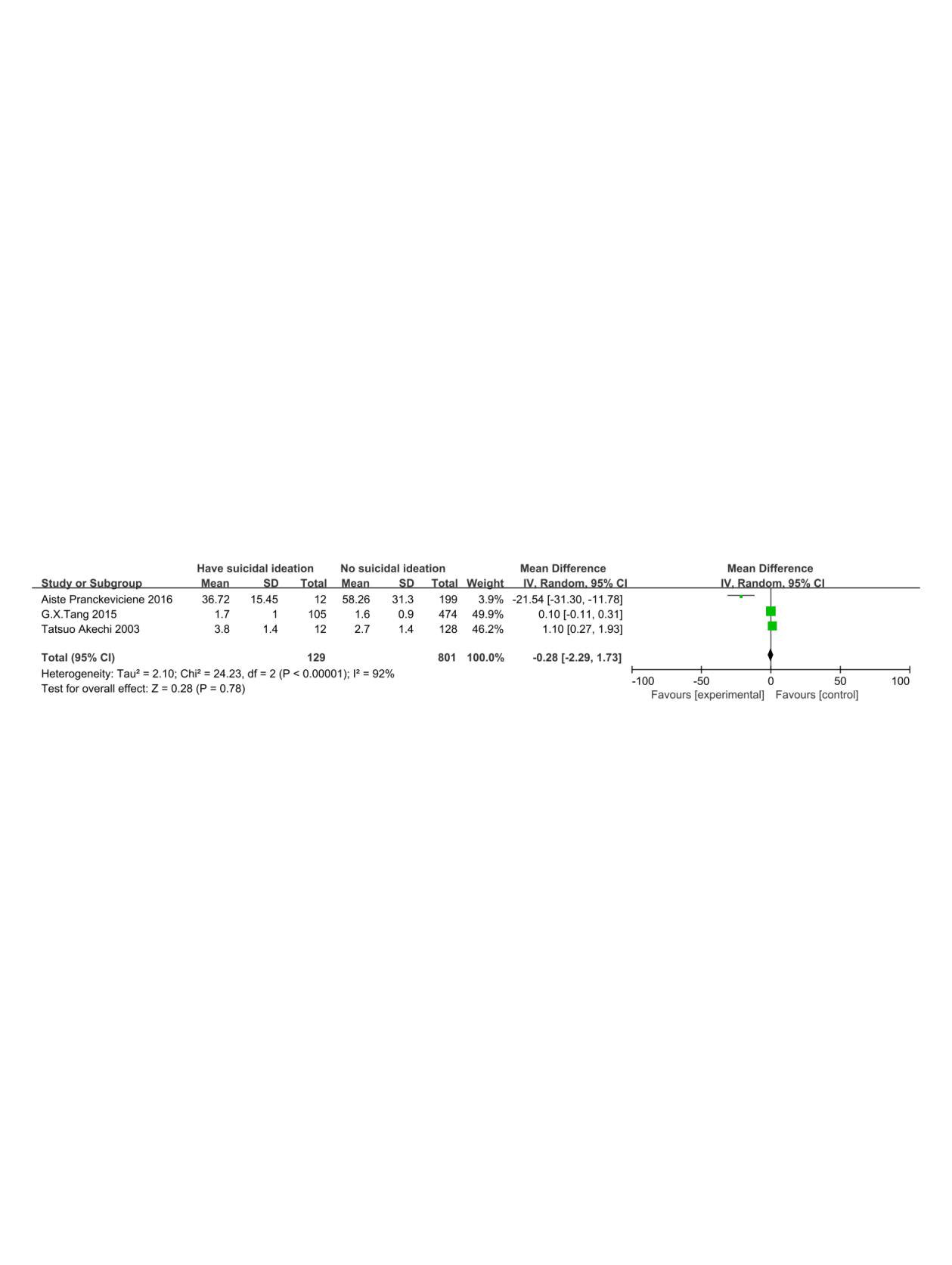
**

Appendix-1 Pain (MD)

**
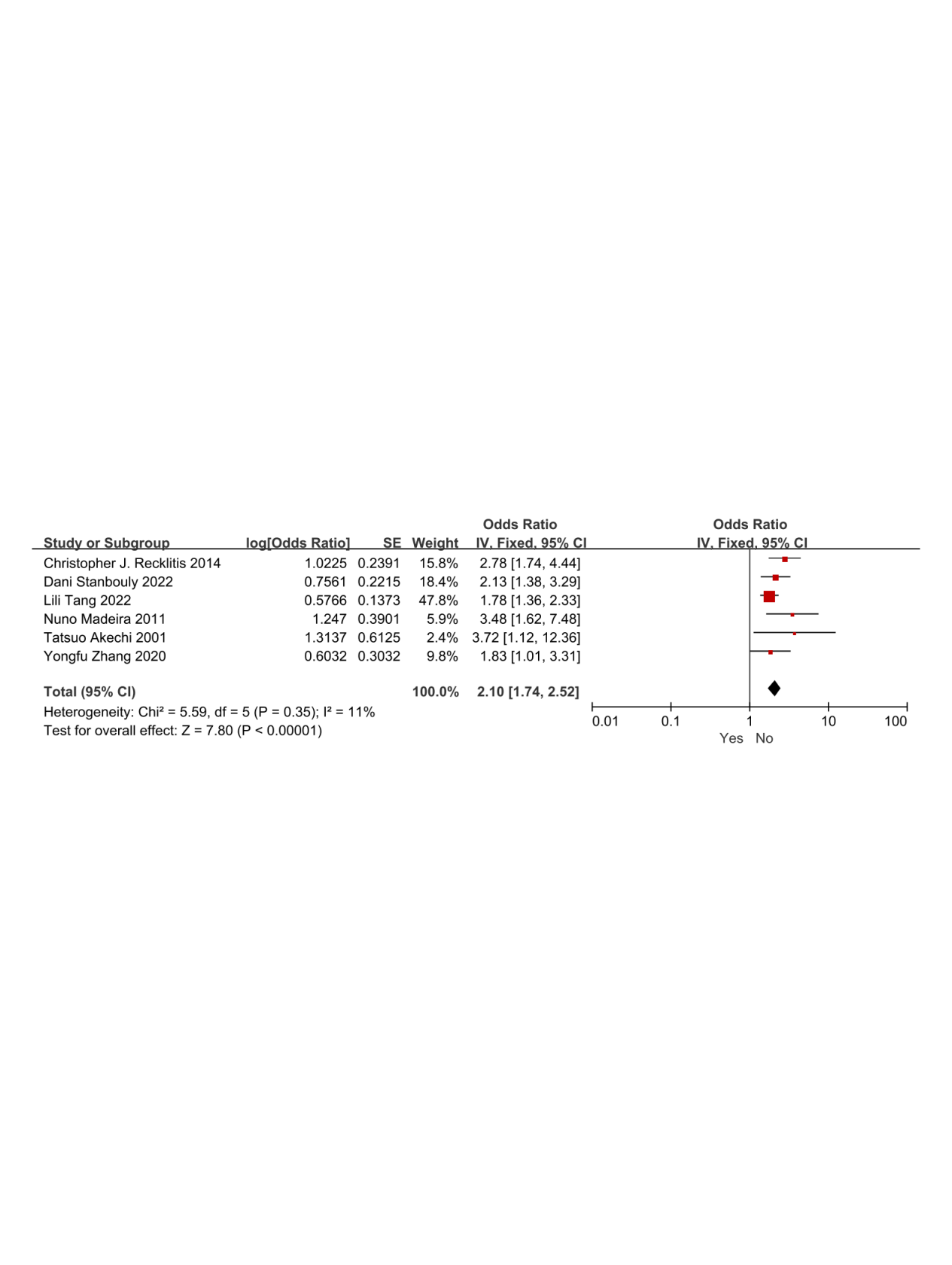
**

Appendix-1 Pain (OR)

**
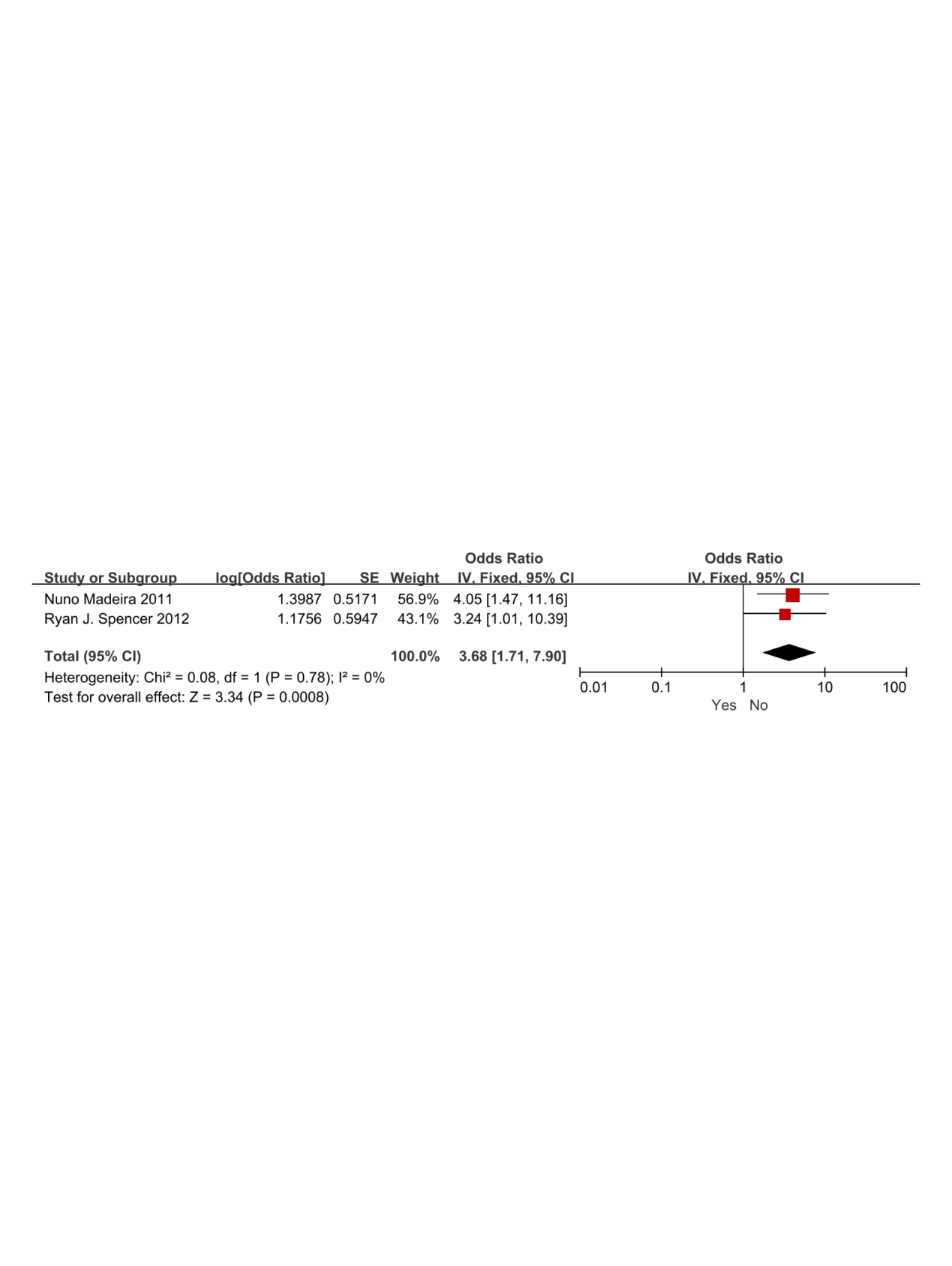
**

Appendix-1 Panic disorder

**
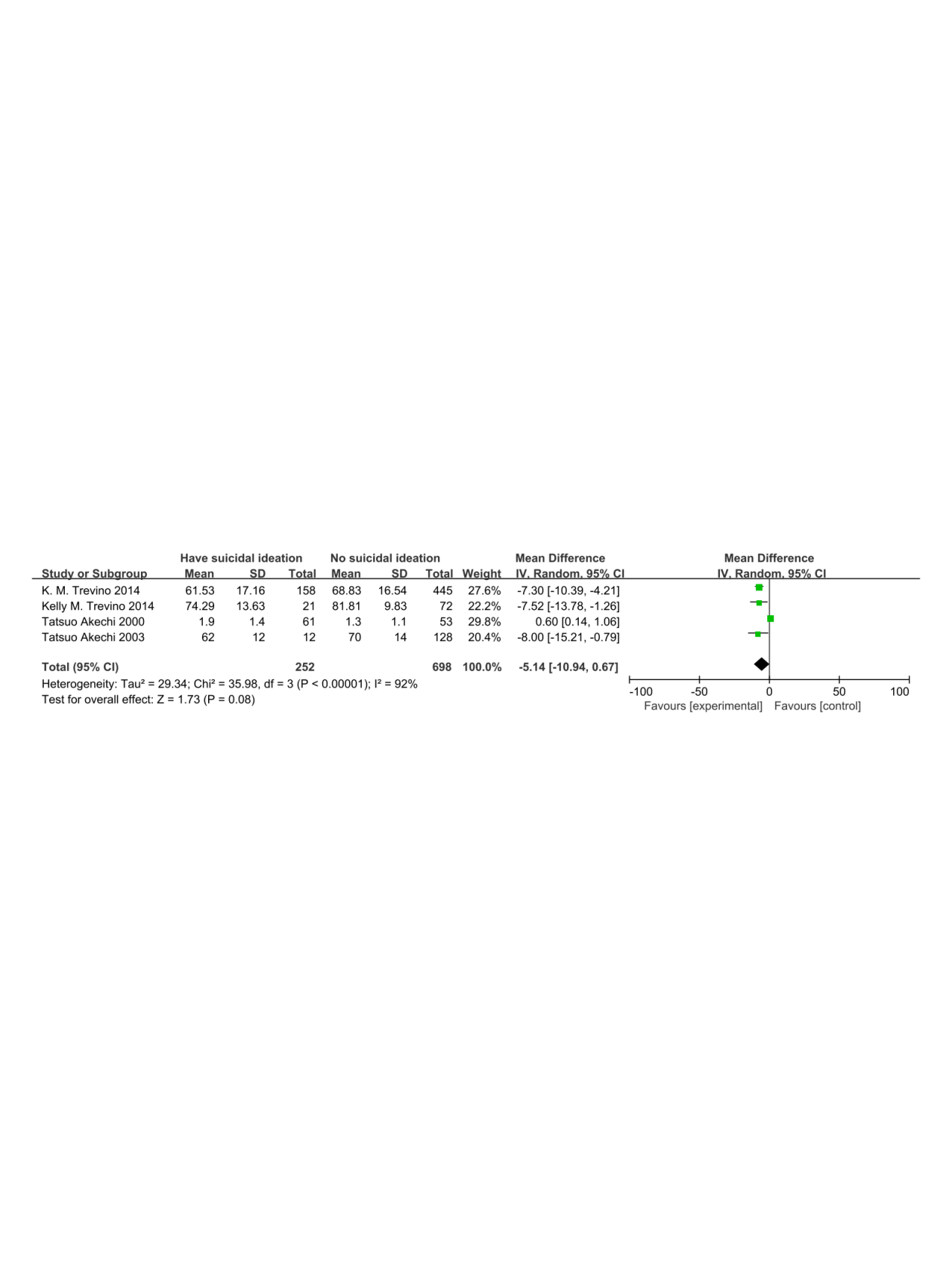
**

Appendix-1 Performance status (MD)

**
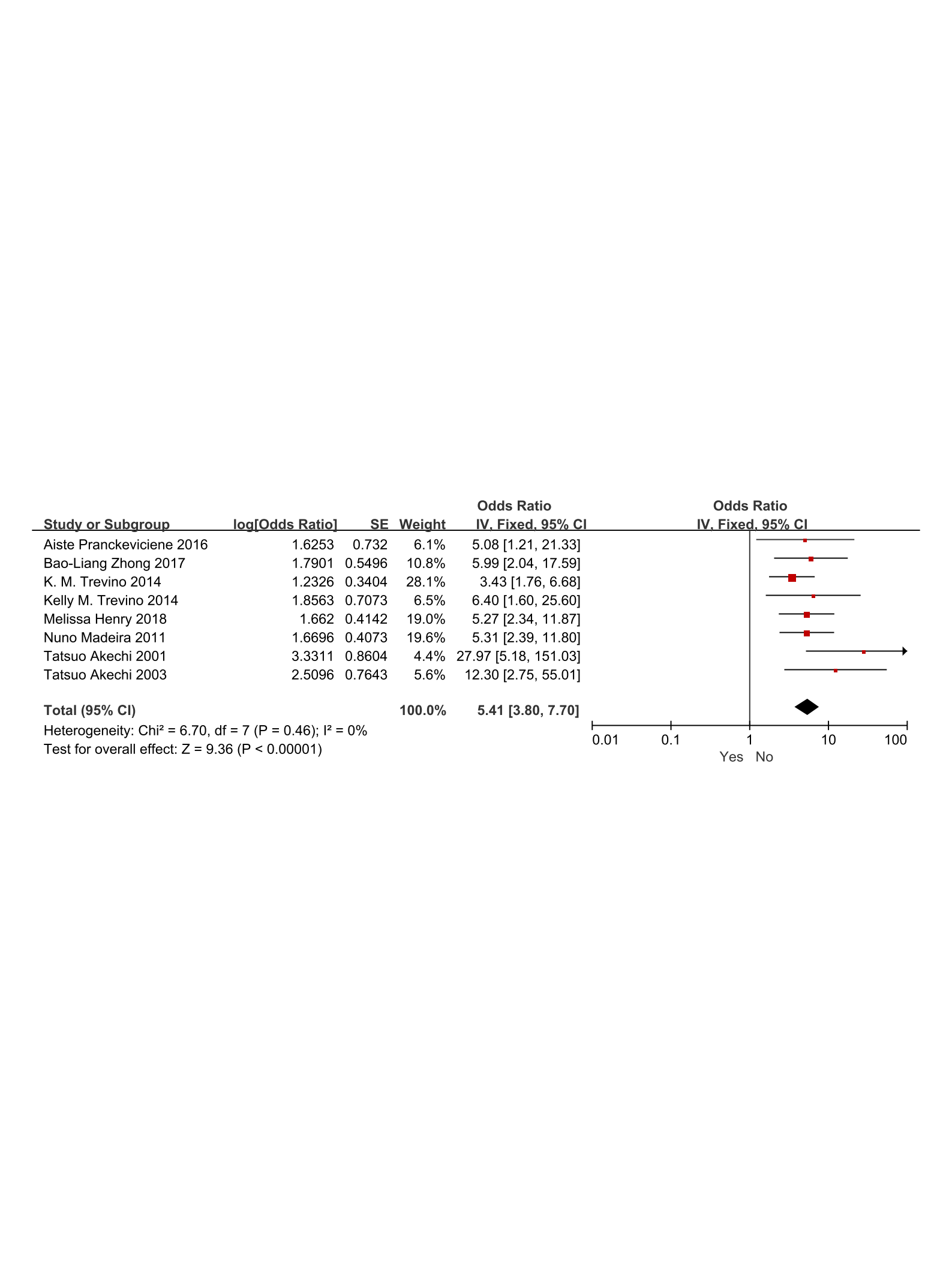
**

Appendix-1 Psychiatric illness history

**
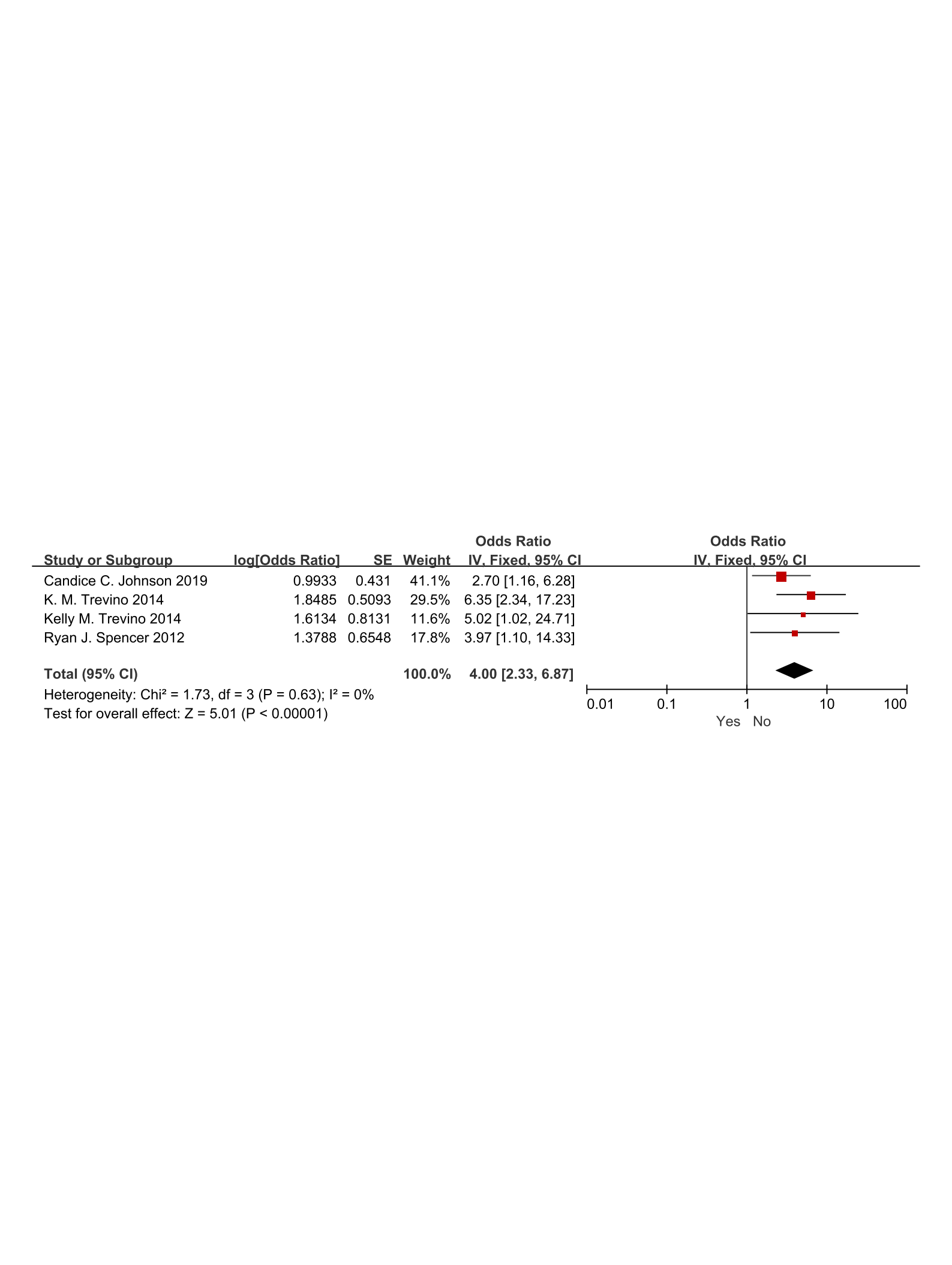
**

Appendix-1 Post-traumatic stress disorder

**
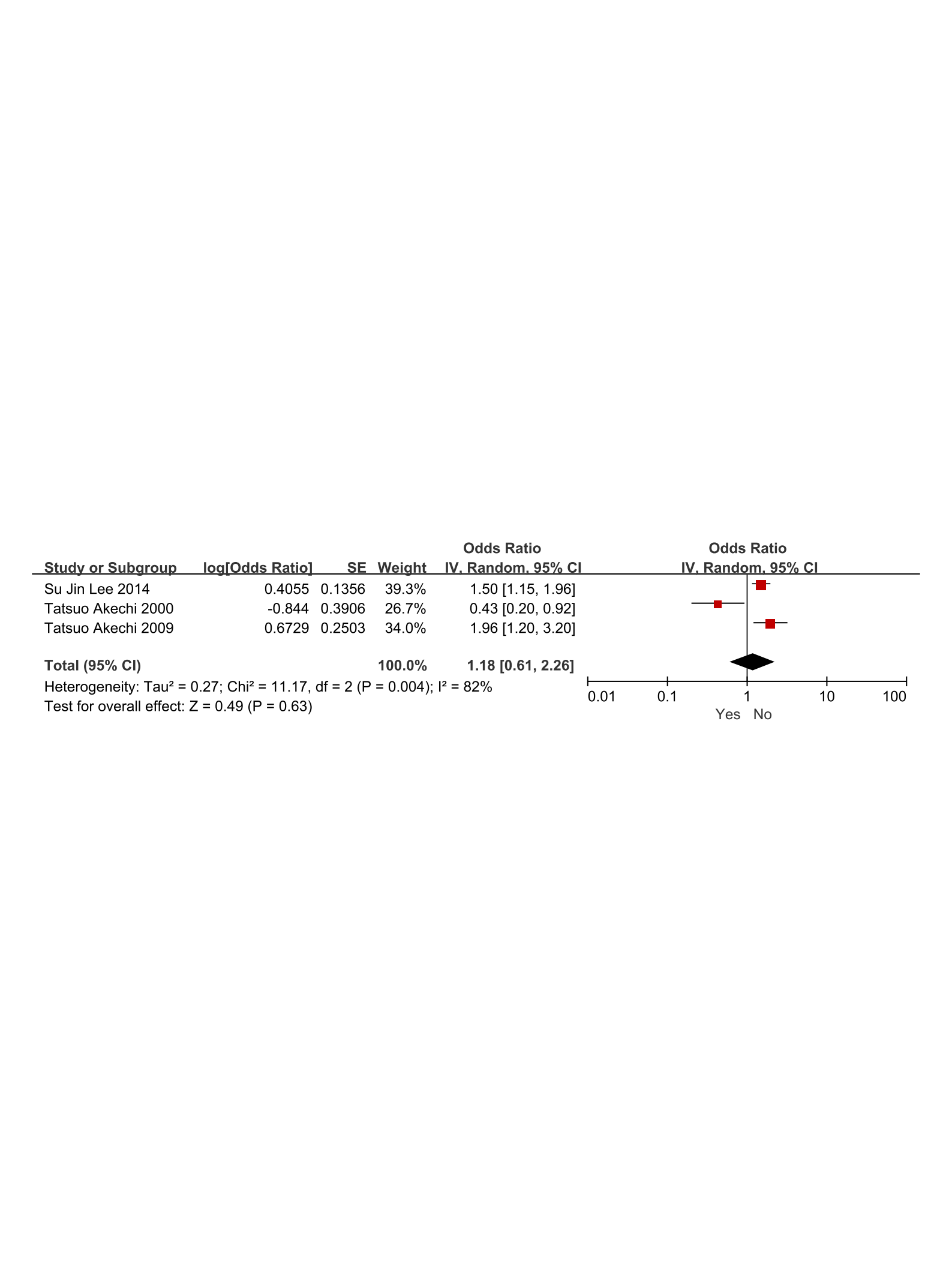
**

Appendix-1 Recurrence

**
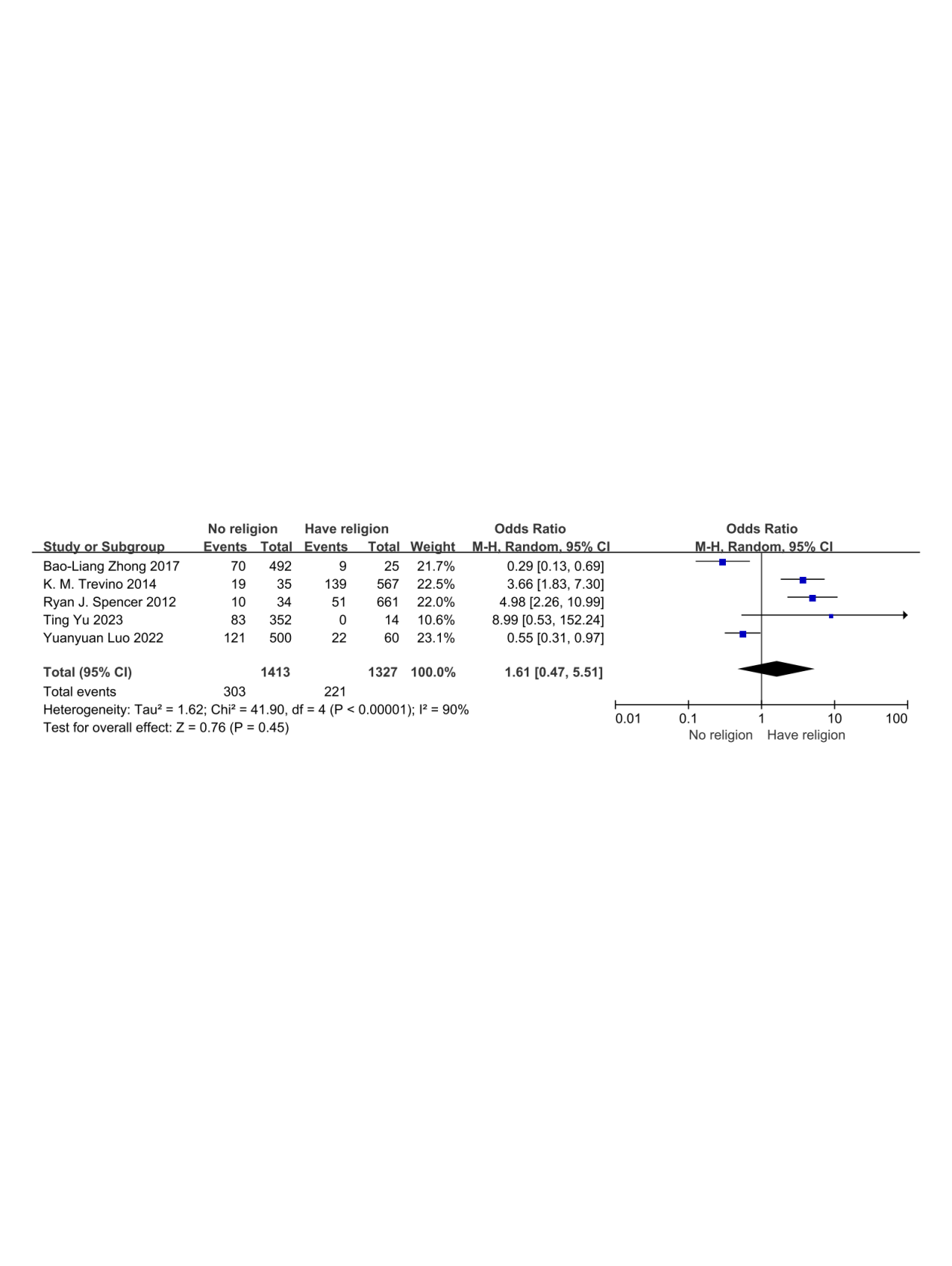
**

Appendix-1 Religion

**
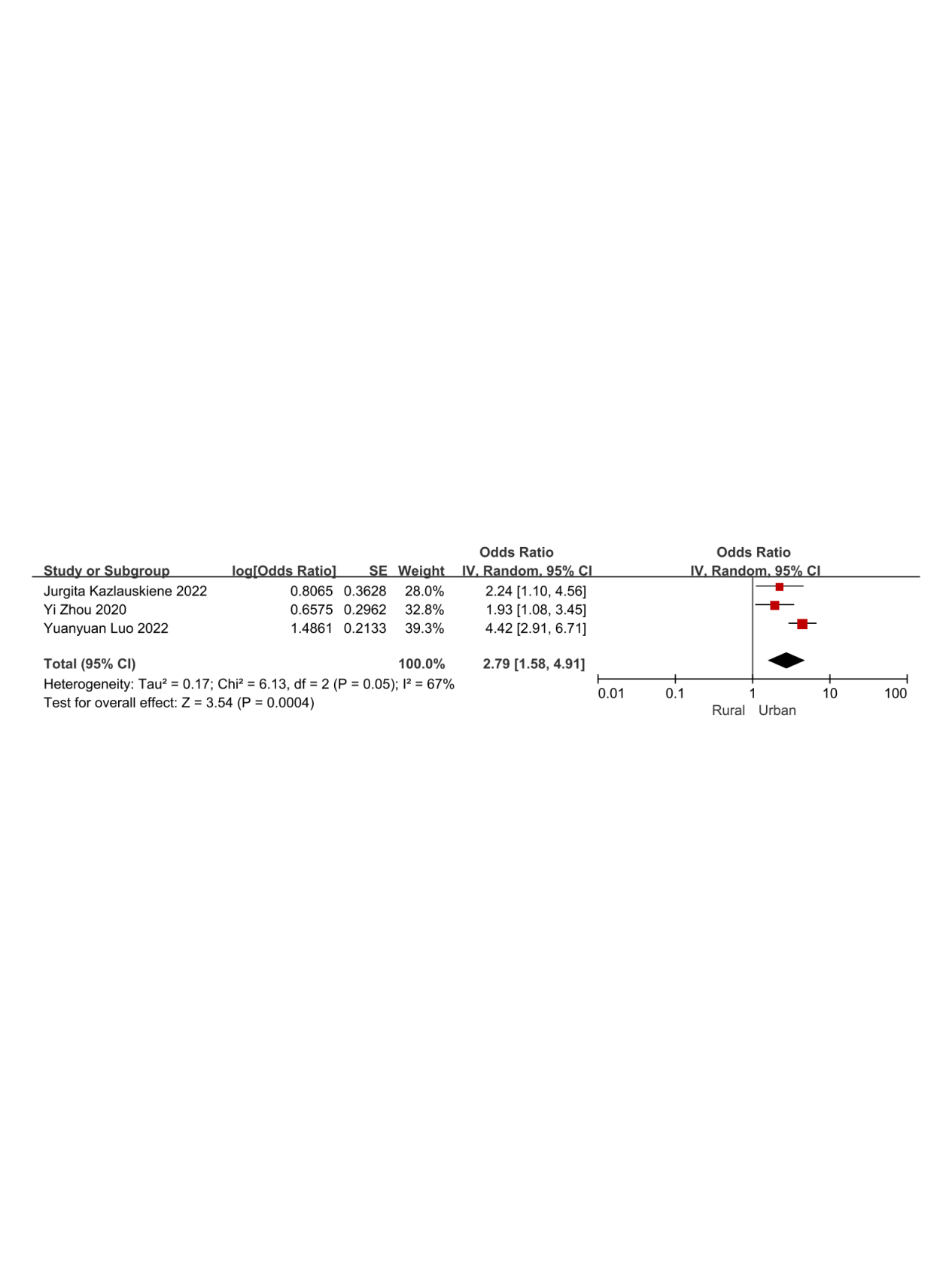
**

Appendix-1 Residence (rural vs. urban)

**
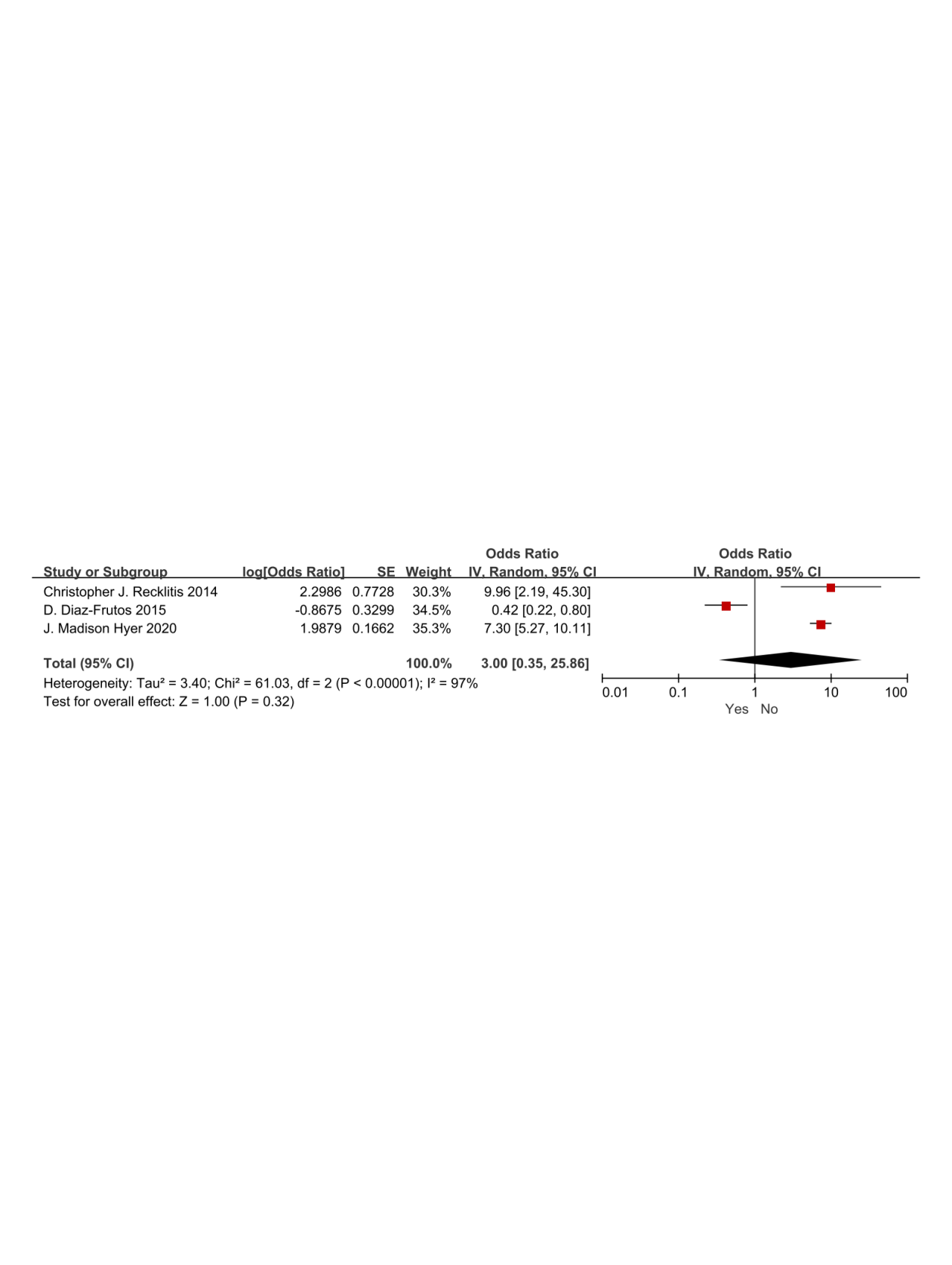
**

Appendix-1 Bipolar/schizophrenia

**
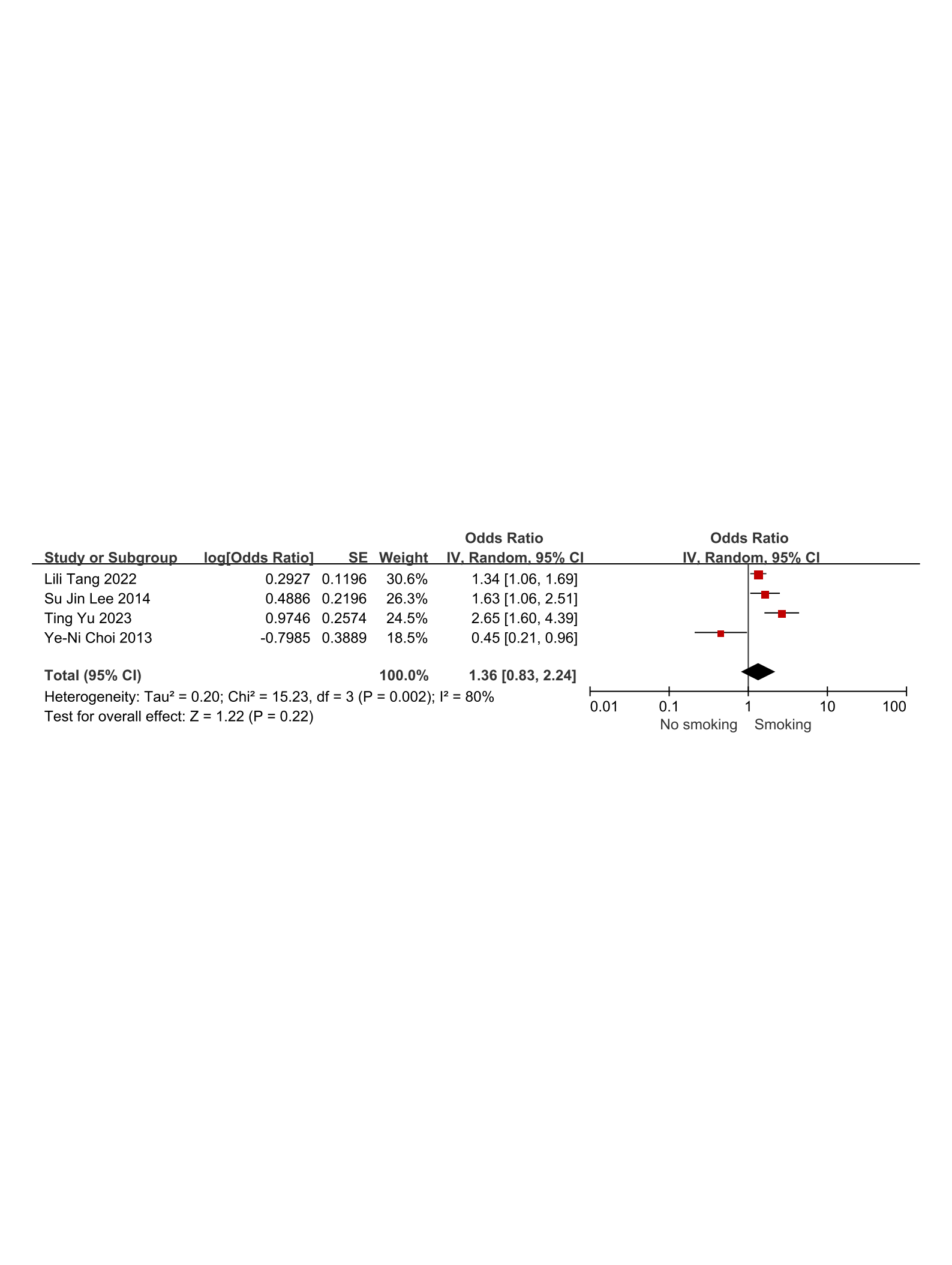
**

Appendix-1 Smoking

**
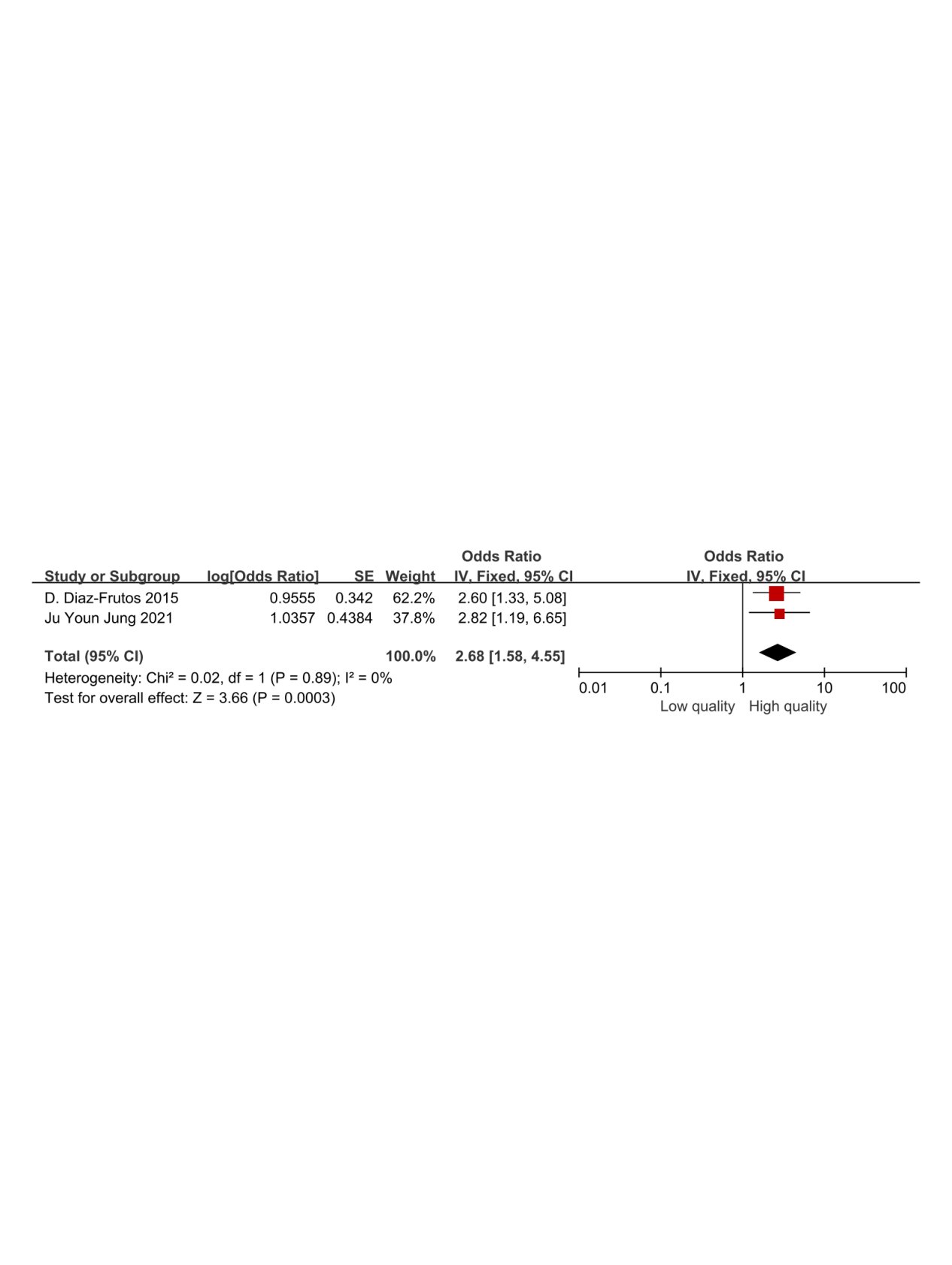
**

Appendix-1 Social functioning (low vs. high)

**
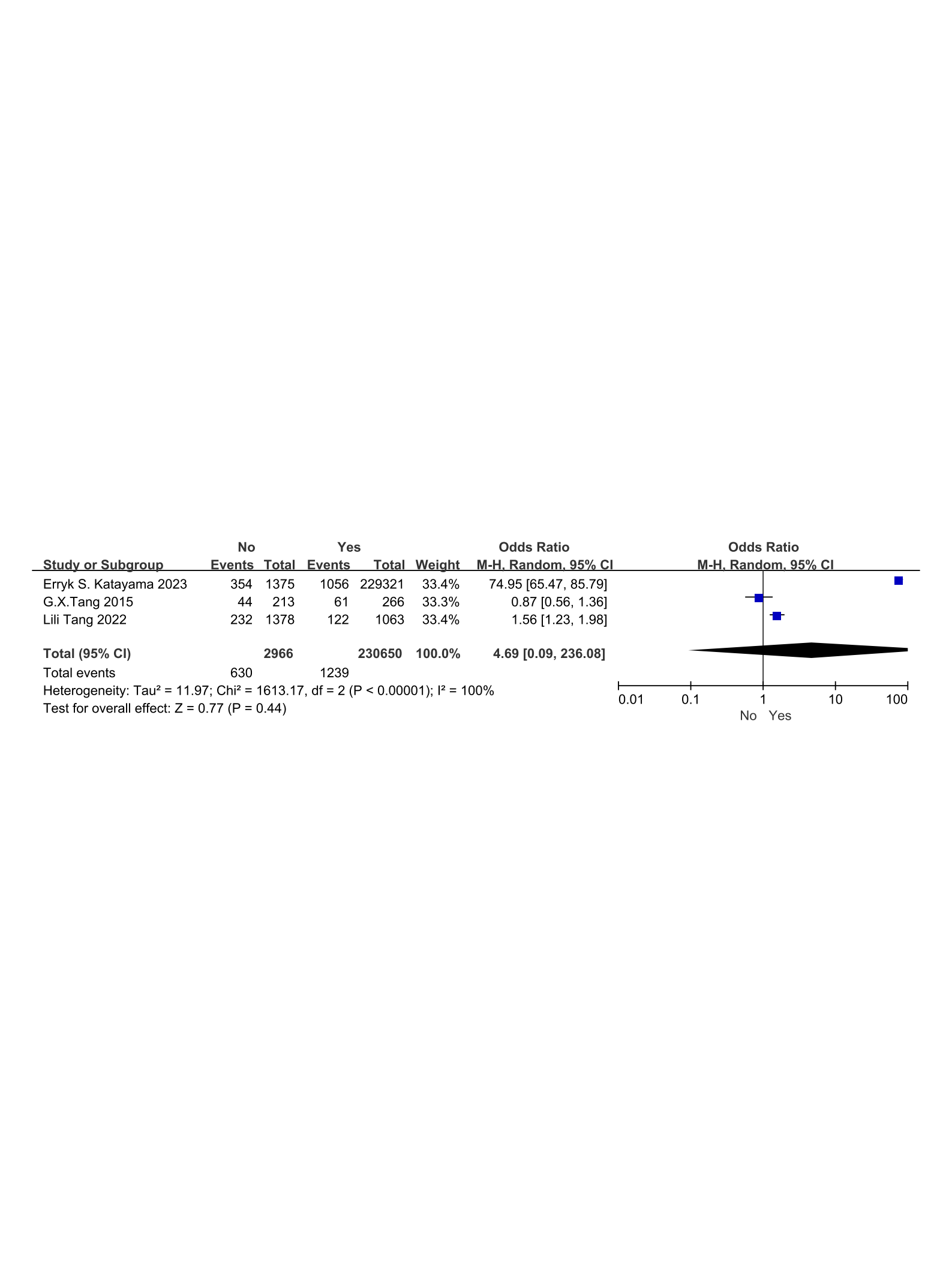
**

Appendix-1 Surgery history

**
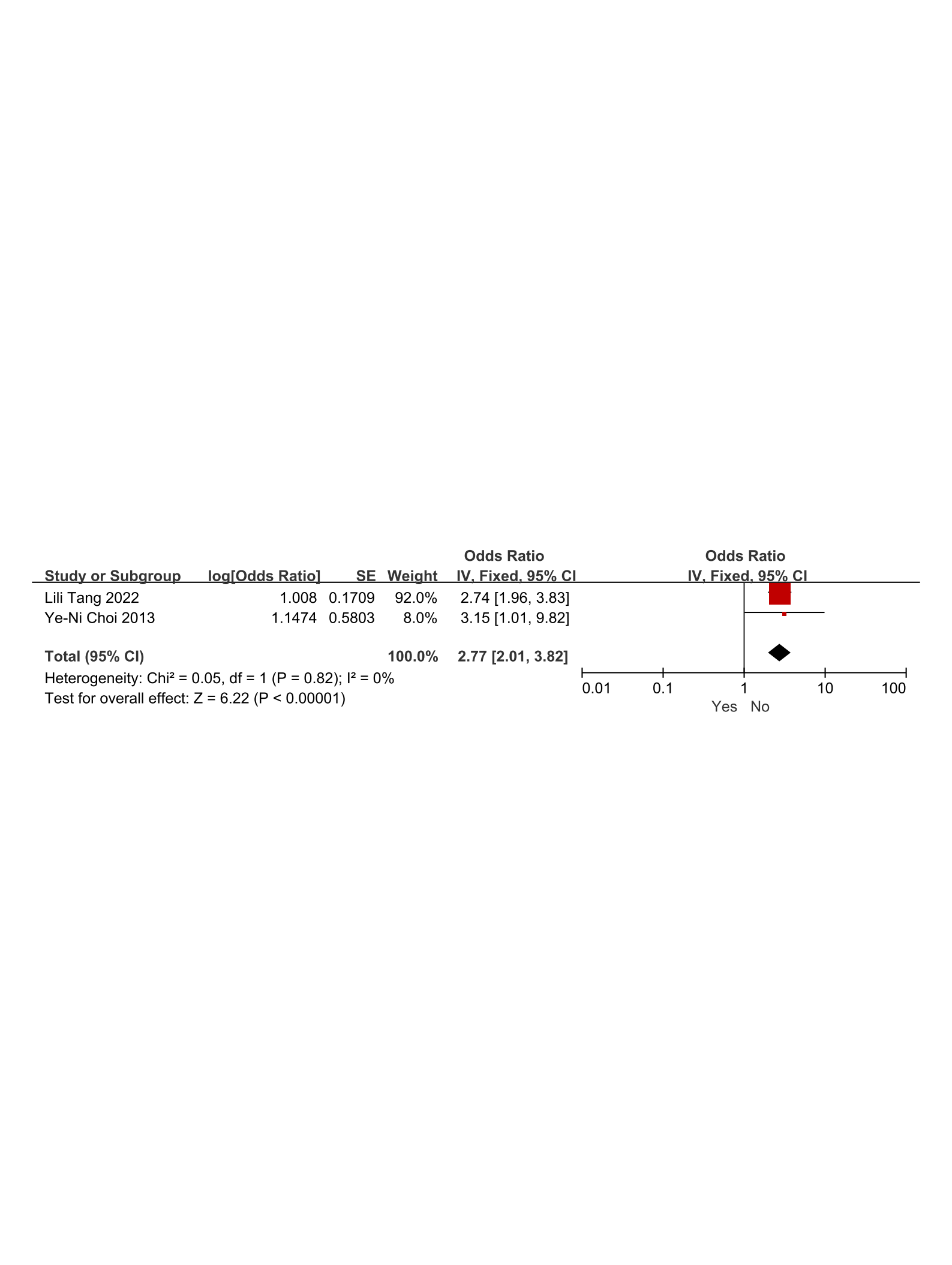
**

Appendix-1 Vomiting

**Supplementary Appendix -2:** Funnel plot

**
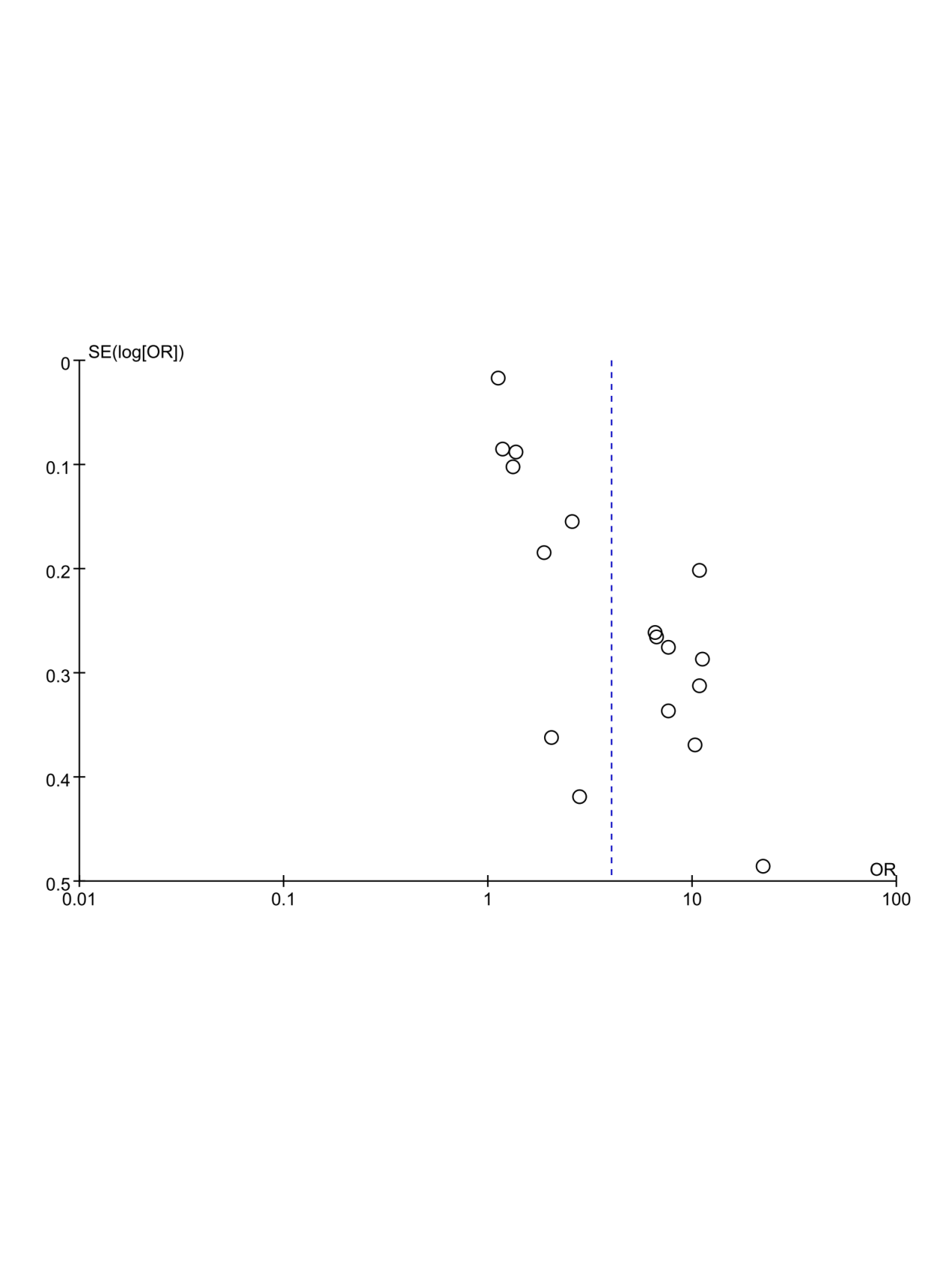
**

Appendix-2 Depression (OR)
